# Epidemiological Evidence Between Variants in Matrix Metalloproteinases-2, -7, and -9 and Cancer Risk

**DOI:** 10.3389/fonc.2022.856831

**Published:** 2022-04-28

**Authors:** Chenglu Huang, Suqin Xu, Zhilin Luo, Dong Li, Rui Wang, Tianhu Wang

**Affiliations:** ^1^ Department of Thoracic Surgery, The Third Affiliated Hospital of Chongqing Medical University, Chongqing, China; ^2^ Department of Radiology, Chongqing University Cancer Hospital, Chongqing, China

**Keywords:** matrix metalloproteinases, variant, meta-analysis, gene, cancer

## Abstract

**Background:**

Matrix metalloproteinases (*MMP*s), a kind of proteases, have a critical function in cancer occurrence, invasion, and migration. *MMP* gene variants (e.g., *MMP-2*, *MMP-7*, and *MMP-9*) can affect the biological functions of these enzymes and lead to the occurrence and progression of cancer, which has become a hot topic in recent years, but the corresponding results are still controversial. In this context, here, the meta-analysis was conducted for assessing the relations of variants in *MMP-2*, *MMP-7*, and *MMP-9* with the risk of various cancers.

**Methods:**

PubMed, Web of Science, and Medline were systemically searched, and data were extracted from all eligible studies so as to investigate the susceptibility of *MMP-2*, *MMP-7*, and *MMP-9* to different types of cancers. The association between a variant in *MMP* and cancer susceptibility was analyzed through odds ratios (ORs) as well as 95% CIs. The Venice criteria and false-positive report probability (FPRP) were adopted to evaluate epidemiological evidence of significant associations discovered.

**Results:**

The associations between the variants of *MMP*s and cancer risk in 36,530 cases and 41,258 controls were found, with 12 associations (*MMP-2* rs243865 with esophageal cancer and lung cancer, *MMP-7* rs11568818 with bladder and cervical cancer, and *MMP-9* rs3918242 with breast cancer) rated as strong associations for cancer risk and 7 and 15 as moderate and weak associations, respectively. These significant associations were mostly found in Asians.

**Conclusions:**

These findings support the relations between variants of *MMP-2*, *MMP-7*, and *MMP-9* and various cancers risk, demonstrating the credibility of these relations.

## Introduction

Cancer accounts for a major cause resulting in global mortality following ischemic heart disease, and the number of death cases and morbidity cases is increasing year by year, thus likely becoming the first in 2060 (1, 2). In previous works, *MMP*s are the most prominent family of proteinases associated with tumorigenesis (3). They are the zn-dependent endopeptidases, which are responsible for degrading basement membrane (BM) and extracellular matrix (ECM), participating in tumor genesis and development (4, 5). Actually, the relationship between these enzymes and tumors is mainly manifested in mediating cell–cell and cell–stromal interactions, thus promoting tumor cell migration and angiogenesis. Here, it should be noted that the remodeling of ECM and BM can be considered an important tumor cell migration and invasion process. *MMP*s are responsible for degrading each BM and ECM protein component and breaking the cancer cell invasion barrier and have important functions in cancer migration and invasion, which have been thus regarded as the major proteases (6–8). According to the substrate and fragment homology, *MMP*s are divided into six categories, namely, collagenase, gelatinase, stroma degrading, stroma lysin, furin-activated *MMP*, and other secreted *MMP*s.

*MMP-2*, *MMP-7*, and *MMP-9* account for the three key components in *MMP* family. *MMP-2* is widely distributed *in vivo* and expressed in most cells including stromal cells, endothelial cells, and epithelial cells, with a relative molecular weight of 72,000, also known as gelatinase A, which can hydrolyze type IV, V, I, and III collagen, laminin, and elastin (9). *MMP*-*7*, which is called matrilysin as well, represents the smallest matrix metalloproteinase due to its lack of a carboxy-terminal heme-binding protein-like domain (10). Active *MMP*-*7* not only degrades ECM but also activates other potential forms of *MMP*s, such as *MMP*-*2* and *MMP*-*9.* As for *MMP*-*9* aka gelatinase B, its precursor can be secreted by monocytes, macrophages, neutrophils, vascular smooth muscle cells, endothelial cells, foam cells, fibroblasts, microglial cells, and tumor cells (11–13). Furthermore, it is activated by enzymatic hydrolysis at or near the 87th amino acid residues, which can hydrolyze various components of BM and ECM, such as collagen IV, thus playing a key role in cancer cell migration and invasion (14).

As early as 2002, Yu et al. discovered in their case–control research that *MMP-2* rs243865 was associated with a higher lung cancer (LC) susceptibility (odds ratio (OR) = 2.15, 95% CI = 1.70–2.71, *p* < 0.05) in Asian populations (15); however, in 2019, Chen et al. reported in their case–control research in the Asian populations that *MMP-2* rs243865 reduced LC susceptibility (OR = 0.54, 95% CI = 0.41–0.72, *p* < 0.05) (16). Moreover, in 2015, Zhang et al. found that *MMP-2* rs243865 had a decreased risk of esophageal squamous cell carcinoma (ESCC) (OR = 0.32, 95% CI = 0.10–0.89, *p* = 0.02) in a case–control study (17); interestingly, Eftekhary et al. revealed that *MMP-2* rs243865 had no association with risk of ESCC among the Asian populations (OR = 0.86, 95% CI = 0.39–1.93, *p* = 0.718) (18). Apart from that, in 2010, Peng et al. pointed out in their meta-analysis that *MMP-2* rs243865 was not related to colorectal cancer (CRC) (19). On the other hand, in 2015, according to Wu et al., they discovered in their meta-analysis that *MMP-2* rs243865 was a risk factor for CRC susceptibility, especially in Caucasians (20).

Although the relations between *MMP-2*, *MMP-7*, and *MMP-9* and various tumors risk had been demonstrated in previous studies, the conclusions were inconsistent. Therefore, in order to obtain more accurate conclusions, this integrative meta-analysis for evaluating the relations of *MMP-2*, *MMP-7*, and *MMP-9* variants with the risk of cancer was conducted.

## Materials and Methods

### Literature Search

PubMed, Embase, and Web of Science were searched for identifying related articles from inception to June 20, 2021, by adopting the following terms: (“tumor” or “malignant” or “malignancy” or “neoplasm” or “neoplasia” or “oncology” or “cancer” or “carcinoma” or “adenocarcinoma”), (“variant” or “variation” or “genotype” or “mutation” or “rs” or “polymorphism” or “single nucleotide polymorphism” or “SNP”), and (“matrix metalloproteinase” or “matrix metalloproteinases” or “MMP” or “MMPs” or “metalloproteinases” or “collagenase” or “gelatinase” or “matrilysin”). Furthermore, reference lists were also manually retrieved to discover eligible articles.

### Criteria for Selection

Studies were selected by the following criteria: a) investigating associations between variants in *MMP-2*, *MMP-7*, and *MMP-9* and cancer risk by cohort, or case–control or cross-sectional studies in humans; b) being published in English; and c) providing case and control numbers, or available allele distribution and/or genotype number when necessary. Studies conforming to the following criteria were eliminated: a) not enough data and b) being in the form of meta-analyses, review articles, abstracts, editorials, letters to the editor, case reports, guidelines for management, and animal studies.

### Data Extraction

Two authors (CH and SX) were responsible for data extraction; any disagreement between them was settled through mutual negotiation. The information extracted included the first author, country, race, publication year, tumor type, genetic variant, gene name, case and control numbers, and genotype distribution frequencies in cases and controls. In our study, the data of Asians and Caucasians, as well as those of different races in three genetic models, were mainly analyzed. For the mutation pattern of a genetic variant, https://www.ncbi.nlm.nih.gov/snp/ was browsed for confirmation.

### Statistical Analysis

All data were obtained by Stata software, version 12.0 (Stata, College Station, TX, USA). The three genetic models were analyzed comprehensively, and ethnic subgroup analysis was performed where necessary. *I* ^2^ statistics and Cochran’s *Q* test were applied in evaluating data heterogeneities from different articles, while heterogeneity was classified by *I* ^2^ value into three levels, ≤25%, 25%–50%, and ≥50%, which stood for little, moderate, and large heterogeneities, respectively. In addition, *P_Q_* < 0.1 indicated that a random-effects model must be adopted; or else, a fixed-effect model should be utilized. In addition, the robustness of the ORs with significant analyses was evaluated by sensitivity analysis, such as the first published study and studies deviated from the Hardy–Weinberg equilibrium (HWE) among controls. The small-study effect was analyzed by Egger’s test, whereas potential publication bias by Begg’s test (*p* < 0.1 is usually considered evidence for significant evidence of small-study effect or publication bias).

### Evaluation of Cumulative Evidence

The Venice criteria were adopted for evaluating epidemiological evidence of obvious associations obtained from meta-analyses, which were graded as weak, moderate, and strong according to the replication of association, amount of evidence, and protection from bias. The A, B, or C grade was given according to the aforementioned criteria. Replication of association was evaluated through heterogeneity statistics, which was classified as grade A, B, or C depending on *I* ^2^ value (≤25%, 25%–50%, or ≥50%, respectively). The amount of evidence (grade A, B, or C) was evaluated through the overall genotype or allele number of control and case groups. (grade A: large scale evidence, minor genetic groups (alleles or genotypes) in cases and controls >1,000; grade B: moderate amount of evidence, minor genetic groups in cases and controls between 100 and 1,000; and grade C: little evidence, minor genetic groups in cases and controls <100). Protection from bias was mainly measured through bias tests and sensitivity analysis, like a single study (dataset), or the first published study, or studies that deviated from the HWE among controls. Grade A indicated no observable bias, and bias was unlikely to explain the presence of the association. Grade B suggested that there was considerable missing information on the identification of evidence, while grade C indicated that there was bias explaining the association. According to the Venice criteria, cumulative epidemiologic creditability for significant association was rated as a strong association if all three grades were A, moderate if a combination of A or B, and weak if any grade was C.

The presence of significant association that might be eliminated as the false-positive result by the false-positive report probability (FPRP) test was analyzed (21). Furthermore, the FPRP with a cutoff value of 0.20 and a prior probability concerning the significant association of 0.05 was calculated. As for FPRP values, <0.05, 0.05–0.20, and >0.20 indicated strong, moderate, and weak creditability of true association, respectively. Later, the FPRP test was conducted to reassess the credibility of the Venice criteria. In the case of strong evidence for true association evidenced by the FPRP test, cumulative evidence was upgraded from moderate to strong or from weak to moderate. In addition, in the case of weak evidence of true association, cumulative evidence was downgraded from strong to moderate or from moderate to weak. If the evidence for a true association was moderate, 0.05 < FPRP < 0.20, the cumulative evidence was neither upgraded nor downgraded.

## Results

### Characteristics of Eligible Studies

**Figure 1:**
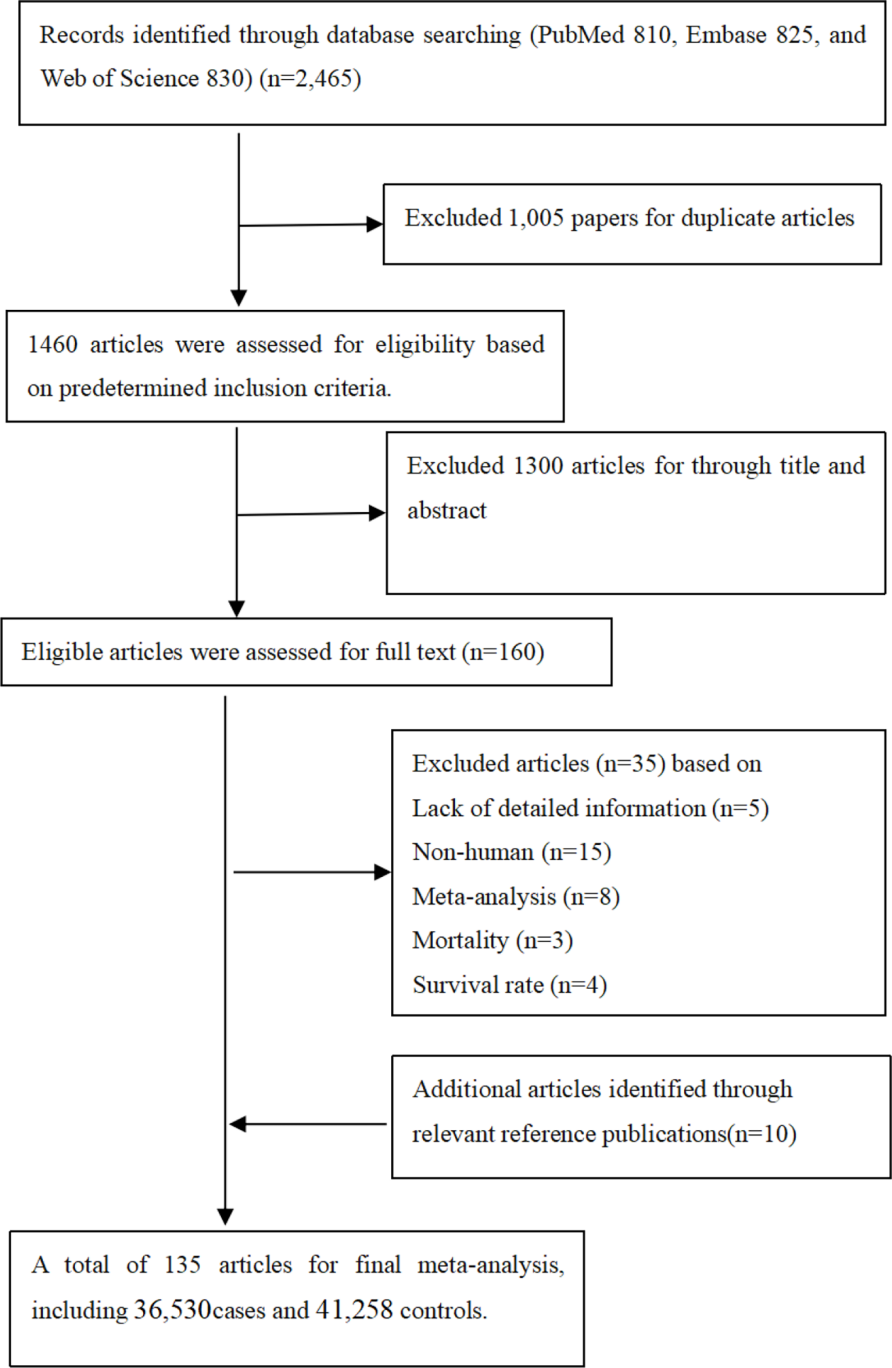
Flow diagram of search strategy and study selection.

It was observed from **Figure 1** that PubMed, Embase, and Web of Science databases were systemically searched for identifying related articles, and altogether, 135 studies were obtained. Among them, 23 articles were eliminated through abstract and keyword reading, while 12 articles were eliminated through full-text reading. Furthermore, 10 articles were selected from the references. Finally, 135 articles on *MMP-2*, *MMP-7*, and *MMP-9* polymorphisms related to cancer risk were included in the meta-analysis, among which 36,530 were cases and 41,258 were controls. Other than that, **Supplementary Table S2** shows basic characteristics of articles, including the first author, the publication year, cases and controls, cancer and genotype, ethnicity, and the rs number. In addition to that, in these papers, the relations between *MMP-2*, *MMP-7*, and *MMP-9* polymorphisms and the risk of a variety of cancers were evaluated.

### Main Meta-Analyses

Meta-analyses were conducted for assessing the relations among variants in *MMP-2*, *MMP-7*, and *MMP-9* and cancer risk. These results are shown in **Table 1**. There were three variants remarkably related to cancer risk, including *MMP-2* rs243865, *MMP-7* rs11568818, and *MMP-9* rs3918242. To be specific, in our research, a significant association between *MMP-2* rs243865 and esophageal cancer risk in Asians was demonstrated (allelic model, OR = 0.751, 95% CI = 0.643–0.877, *p* < 0.001; dominant model, OR = 0.723, 95% CI = 0.607–0.862, *p* < 0.001). Furthermore, it was also revealed that *MMP-2* rs243865 had significant association with LC incidence among the overall populations under the allelic and dominant models (allelic model, OR = 0.654, 95% CI = 0.507–0.844, *p* = 0.001; dominant model, OR = 0.613, 95% CI = 0.457–0.823, *p* = 0.001). Apart from that, *MMP-2* rs243865 in the recessive, dominant, and allelic models showed obvious relations with LC incidence among Asian populations (allelic model, OR = 0.534, 95% CI = 0.468–0.610, *p* < 0.001; dominant model, OR = 0.484, 95% CI = 0.417–0.561, *p* < 0.001; recessive model, OR = 0.616, 95% CI = 0.385–0.985, *p* = 0.043). *MMP-2* rs243865 was significantly related to nasopharyngeal cancer (NPC) risk among the Asian populations by the dominant model (OR = 0.686, 95% CI = 0.492–0.957, *p* = 0.026). With regard to prostate cancer (PCa), *MMP-2* rs243865 was significantly related to PCa incidence among the overall populations in the dominant model (OR = 1.365, 95% CI = 1.094–1.703, *p* = 0.006). Furthermore, *MMP-2* rs243865 was dramatically related to PCa incidence among the Asian populations (dominant model, OR = 1.657, 95% CI = 1.207–2.276, *p* = 0.002; allelic model, OR = 1.480, 95% CI = 1.131–1.936, *p* = 0.004).

**Table 1:** Significant associations between variants in the *MMP-2*, *MMP-7*, and *MMP-9* and cancer risk.

Gene	Variant	Alleles	Cancer site	Ethnicity	MAF	Number evaluation		Risk of Meta-Analysis						Venice criteria	FPRP values	Credibility of evidence
						Studies	Sample size (cases/controls)	Genetic models	Effect model	OR (95% CI)	*p*-Value	*I*2	PQ			
MMP2	rs243865	TvsC	Esophageal	Asian	0.1565	4	2,850 (1,157/1,693)	Allelic	Fixed	0.751 (0.643–0.877)	<0.001	41.4	0.163	BBA	0.006	Strong
MMP2	rs243865	TvsC	Esophageal	Asian	0.1565	4	2,850 (1,157/1,693)	Dominant	Fixed	0.723 (0.607–0.862)	<0.001	46.9	0.13	BBA	0.007	Strong
MMP2	rs243865	TvsC	Lung	Overall	0.1679	5	4,734 (2,199/2,535)	Allelic	Random	0.654 (0.507–0.844)	0.001	72.1	0.006	ACC	0.045	Moderate
MMP2	rs243865	TvsC	Lung	Overall	0.1679	5	4,734 (2,199/2,535)	Dominant	Random	0.613 (0.457–0.823)	0.001	73.8	0.004	ACC	0.07	Weak
MMP2	rs243865	TvsC	Lung	Asian	0.1667	3	4,254 (1,909/2,345)	Allelic	Fixed	0.534 (0.468–0.610)	<0.001	0.0	0.837	AAA	5.60083E−07	Strong
MMP2	rs243865	TvsC	Lung	Asian	0.1667	3	4,254 (1,909/2,345)	Dominant	Fixed	0.484 (0.417–0.561)	<0.001	0.0	0.864	AAA	2.79371E−10	Strong
MMP2	rs243865	TvsC	Lung	Asian	0.1667	3	4,254 (1,909/2,345)	Recessive	Fixed	0.616 (0.385–0.985)	0.043	0.0	0.944	CAC	0.688	Weak
MMP2	rs243865	TvsC	Nasopharyngeal	Asian	0.1083	3	2,946 (1,381/1,565)	Dominant	Random	0.686 (0.492–0.957)	0.026	63.0	0.067	BCC	0.47	Weak
MMP2	rs243865	TvsC	Prostate	Overall	0.2044	6	1,433 (699/734)	Dominant	Fixed	1.365 (1.094–1.703)	0.006	7.1	0.371	BAA	0.122	Moderate
MMP2	rs243865	TvsC	Prostate	Asian	0.1535	3	732 (341/391)	Allelic	Fixed	1.480 (1.131–1.936)	0.004	0.0	0.868	BAA	0.13	Moderate
MMP2	rs243865	TvsC	Prostate	Asian	0.1535	3	732 (341/391)	Dominant	Fixed	1.657 (1.207–2.276)	0.002	0.0	0.962	BAA	0.114	Moderate
MMP7	rs11568818	CvsT	Bladder	Overall	0.291	4	2,377 (1,169/1,208)	Allelic	Fixed	1.204 (1.055–1.374)	0.006	0.0	0.405	AAA	0.1	Strong
MMP7	rs11568818	CvsT	Bladder	Overall	0.291	4	2,377 (1,169/1,208)	Recessive	Fixed	1.538 (1.198–1.974)	0.001	0.0	0.696	BAA	0.032	Strong
MMP7	rs11568818	CvsT	Bladder	Asian	0.2651	3	1,938 (929/1,009)	Allelic	Fixed	1.229 (1.056–1.431)	0.008	23.7	0.269	AAA	0.131	Strong
MMP7	rs11568818	CvsT	Bladder	Asian	0.2651	3	1,938 (929/1,009)	Recessive	Fixed	1.560 (1.166–2.087)	0.003	0.0	0.494	BAA	0.116	Moderate
MMP7	rs11568818	CvsT	Cervical	Asian	0.2896	3	1,179 (597/582)	Allelic	Fixed	1.372 (1.148–1.640)	0.001	0.0	0.583	BAA	0.012	Strong
MMP7	rs11568818	CvsT	Cervical	Asian	0.2896	3	1,179 (597/582)	Dominant	Fixed	1.381 (1.088–1.753)	0.008	52.8	0.12	BCC	0.168	Weak
MMP7	rs11568818	CvsT	Cervical	Asian	0.2896	3	1,179 (597/582)	Recessive	Fixed	1.664 (1.175–2.357)	0.004	52.7	0.121	BCA	0.22	Weak
MMP7	rs11568818	CvsT	Colorectal	Asian	0.1292	5	2,214 (1,045/1,169)	Allelic	Fixed	0.771 (0.629–0.945)	0.012	0.0	0.566	BAA	0.202	Weak
MMP7	rs11568818	CvsT	Colorectal	Asian	0.1292	5	2,214 (1,045/1,169)	Recessive	Fixed	0.450 (0.256–0.790)	0.005	12.6	0.285	CAC	0.546	Weak
MMP9	rs3918242	TvsC	Breast	Overall	0.1967	6	3,316 (1,656/1,660)	Allelic	Fixed	1.281 (1.134–1.447)	<0.001	29.2	0.216	AAA	0.001	Strong
MMP9	rs3918242	TvsC	Breast	Overall	0.1967	6	3,316 (1,656/1,660)	Dominant	Fixed	1.236 (1.065–1.434)	0.005	21.8	0.27	AAC	0.09	Weak
MMP9	rs3918242	TvsC	Breast	Overall	0.1967	6	3,316 (1,656/1,660)	Recessive	Fixed	1.681 (1.279–2.209)	<0.001	0.0	0.723	BAA	0.017	Strong
MMP9	rs3918242	TvsC	Breast	Asian	0.3042	3	1,196 (601/595)	Allelic	Fixed	1.501 (1.263–1.785)	<0.001	0.0	0.731	BAC	0.0002	Moderate
MMP9	rs3918242	TvsC	Breast	Asian	0.3042	3	1,196 (601/595)	Dominant	Fixed	1.526 (1.207–1.930)	<0.001	0.0	0.628	BAA	0.018	Strong
MMP9	rs3918242	TvsC	Breast	Asian	0.3042	3	1,196 (601/595)	Recessive	Fixed	1.710 (1.262–2.317)	0.001	0.0	0.917	BAA	0.049	Strong
MMP9	rs3918242	TvsC	Gastric	Asian	0.2385	3	1,128 (539/589)	Recessive	Fixed	1.612 (1.000–2.598)	0.05	0.0	0.551	CAA	0.712	Weak
MMP9	rs3918242	TvsC	Hepatocellular	Overall	0.1589	3	1,280 (657/623)	Recessive	Fixed	1.973 (1.068–3.645)	0.03	48.1	0.165	CBA	0.749	Weak
MMP9	rs3918242	TvsC	Lung	Overall	0.141	5	2,980 (1,539/1,447)	Allelic	Random	0.754 (0.570–0.999)	0.049	63.3	0.028	BCA	0.537	Weak
MMP9	rs3918242	TvsC	Lung	Overall	0.141	5	2,980 (1,539/1,447)	Recessive	Fixed	0.355 (0.177–0.712)	0.004	42.1	0.159	CCA	0.639	Weak
MMP9	rs3918242	TvsC	Lung	Caucasian	0.1496	3	1,890 (1,051/839)	Allelic	Fixed	0.798 (0.661–0.962)	0.018	0.0	0.502	BAA	0.276	Weak
MMP9	rs3918242	TvsC	Lung	Caucasian	0.1496	3	1,890 (1,051/839)	Recessive	Fixed	0.257 (0.118–0.561)	0.001	0.0	0.615	CAC	0.595	Weak
MMP9	rs3918242	TvsC	Oral	Overall	0.1406	3	1,545 (770/775)	Allelic	Fixed	1.309 (1.078–1.589)	0.007	26.8	0.255	BAA	0.118	Moderate
MMP9	rs3918242	TvsC	Oral	Overall	0.1406	3	1,545 (770/775)	Recessive	Fixed	3.497 (1.812–6.749)	<0.001	0.0	0.717	CAA	0.383	Weak

Allelics: minor allelic (bold) vs. major allelic. Venice criteria grades are for amount of evidence, replication of the association, and protection from bias. The prior probability of FPRP is 0.05, and the FPRP level of noteworthiness is 0.20.

C, cytosine; T, thymine; OR, odds ratio; MAF, minor allelic frequency in control; FPRP, false-positive report probability.

For *MMP-7*, it was discovered that *MMP-7* rs11568818 was markedly related to bladder cancer susceptibility among the overall populations under the allelic and recessive models (allelic model, OR = 1.204, 95% CI = 1.055–1.374, *p* = 0.006; recessive model, OR = 1.538, 95% CI = 1.198–1.974, *p* = 0.001) instead of the dominant model; however, in Asians, *MMP-7* rs11568818 was obviously related to bladder cancer risk (allelic model, OR = 1.229, 95% CI = 1.056–1.431, *p* = 0.008; recessive model, OR = 1.560, 95% CI = 1.166–2.087, *p* = 0.003). Moreover, the *MMP-7* rs11568818 was evidently associated with a higher cervical cancer (CC) incidence in Asians (allelic model, OR = 1.372, 95% CI = 1.148–1.640, *p* = 0.001; dominant model, OR = 1.381, 95% CI = 1.088–1.753, *p* = 0.008; recessive model, OR = 1.664, 95% CI = 1.175–2.357, *p* = 0.004). In addition, it was also known that *MMP-7* rs11568818 under the recessive and allelic models was noticeably related to CRC incidence among the Asian populations (allelic model, OR = 0.771, 95% CI = 0.629–0.945, *p* = 0.012; recessive model, OR = 0.450, 95% CI = 0.256–0.790, *p* = 0.005).

*MMP-9* rs3918242 was definitely relevant to breast cancer (BC) incidence among the overall populations (allelic model, OR = 1.281, 95% CI = 1.134–1.447, *p* < 0.001; dominant model, OR = 1.236, 95% CI = 1.065–1.434, *p* = 0.005; recessive model, OR = 1.681, 95% CI = 1.279–2.209, *p* < 0.001) and in Asians (allelic model, OR = 1.501, 95% CI = 1.263–1.785, *p* < 0.001; dominant model, OR = 1.526, 95% CI = 1.207–1.930, *p* < 0.001; recessive model, OR = 1.710, 95% CI = 1.262–2.317, *p* = 0.001) under three models. *MMP-9* rs3918242 was remarkably related to gastric cancer (GC) incidence among the Asian populations in the recessive model (OR = 1.612, 95% CI = 1.000–2.598, *p* = 0.05). Additionally, *MMP-9* rs3918242 was dramatically related to hepatocellular cancer (HCC) incidence among the overall populations in the recessive model (OR = 1.973, 95% CI = 1.068–3.645, *p* = 0.03). On the other hand, *MMP-9* rs3918242 was also markedly related to LC incidence in the recessive or allelic model in all populations (allelic model, OR = 0.754, 95% CI = 0.570–0.999, *p* = 0.049; recessive model, OR = 0.355, 95% CI = 0.177–0.712, *p* = 0.004). Furthermore, *MMP-9* rs3918242 was observably connected to LC incidence in the recessive and allelic models in Caucasians (recessive model, OR = 0.257, 95% CI = 0.118–0.561, *p* = 0.001; allelic model, OR = 0.798, 95% CI = 0.661–0.962, *p* = 0.018). Finally, it was revealed that *MMP-9* rs3918242 under the allelic and recessive models was remarkably related to oral cancer incidence among the overall populations (allelic model, OR = 1.309, 95% CI = 1.078–1.589, *p* = 0.007; recessive model, OR = 3.497, 95% CI = 1.812–6.749, *p* < 0.001).

In addition, in the current study, it can be seen that *MMP-2* rs243865 was not obviously related to the risk of certain cancer types in three models, such as BC, bladder cancer, CRC, GC, oral cancer, and lymphoma. *MMP-2* rs1053605 was not markedly related to LC incidence among the overall populations and Caucasians under three models, while *MMP-7* rs11568818 was not related to the risk of certain cancer types in three models, such as BC, GC, and LC. Furthermore, *MMP-9* rs3918242 was not related to the incidence of certain cancer types, like bladder cancer, CRC, and esophageal cancer. *MMP-9* rs17576 was not related to CRC incidence under three models in Asians.

### Cumulative Evidence of Association

The details of the epidemiological evidence for three variants related to cancer risk can be observed in **Table 1**. Firstly, the Venice criteria were followed to evaluate these associations. As for the amount of evidence, 8, 19, and 7 associations were graded as grades A, B, and C, respectively, for further evaluating evidence credibility. With regard to replication of association, 24, 3, and 7 associations were graded as grades A, B, and C, respectively, for further assessment. As for the protection from bias, 25, 0, and 9 associations were graded as grades A, B, and C, respectively, for additional analysis. Five of the associations were rated as strong (*MMP-2* rs243865 among the Asian populations in the dominant and allelic models with LC risk, *MMP-7* rs11568818 in all populations or Asians under the allelic model with bladder cancer risk, and *MMP-9* rs3918242 in all populations under the allelic model with BC risk), 13 associations were rated as moderate (*MMP-2* rs243865 with esophageal cancer risk among the Asian populations in the dominant and allelic models and with PCa risk among the overall populations in the dominant model, as well as among the Asian populations in the dominant and allelic models; in addition, *MMP-7* rs11568818 was associated with bladder cancer risk among the Asian and overall populations in the recessive model with bladder cancer risk, *MMP-7* rs11568818 in Asians under the allelic model with CC risk, and *MMP-7* rs11568818 in Asians under the allelic model with CRC risk; furthermore, *MMP-9* rs3918242 was related to BC risk among Asian populations in the recessive or dominant model and among the overall populations in the recessive model, and *MMP-9* rs3918242 in all populations under the allelic model with oral cancer risk), and 16 associations were rated as weak (*MMP-2* rs243865 among Asian populations in the recessive model and among the overall populations in the dominant or allelic model with LC risk, *MMP-2* rs243865 in Asians under the dominant model with NPC risk, *MMP-7* rs11568818 in Asians under the dominant or recessive model with CC risk and among Asian population in the recessive model with CRC risk, *MMP-9* rs3918242 associated with BC risk among Asian populations in the allelic model and the overall populations in the dominant model, and *MMP-9* rs3918242 related to HCC risk among the overall populations in the recessive model, to GC risk among Asian populations in the recessive model, and to LC risk among Caucasians or the overall populations in the recessive and allelic models, and in all populations under the recessive model with oral cancer risk) based on the Venice criteria.

By calculating FPRP values, the probability that nominally significant variants were truly related to cancer incidence was assessed. Of the above associations with cancer risk, 12 associations had a *p*-value of FPRP less than 0.05, while 10 associations were featured with a *p*-value from 0.05 to 0.2, and the *p*-value of the remaining 12 associations was greater than 0.2. Consequently, the cumulative evidence of association was reassessed. It was strong for *MMP-2* rs243865 with LC and esophageal cancer risk among the Asian populations in the dominant and allelic models. *MMP-7* rs11568818 was related to bladder cancer risk among the Asian populations in the allelic model, bladder cancer risk among the overall populations in the recessive or allelic model, and CC risk in Asians under the allelic model, whereas *MMP-9* rs3918242 was associated with BC risk among the Asian population in the dominant and recessive models and the overall populations in the recessive and allelic models (**Supplementary Figures S1–S12**); it was moderate for *MMP-2* rs243865 in all populations under the allelic model with LC risk, among the overall populations in the dominant model with PCa risk, and the Asian populations in the dominant and allelic models with PCa risk. Moreover, *MMP-7* rs11568818 in Asians under the recessive model was related to bladder cancer risk, while *MMP-9* rs3918242 was relevant to BC risk among the Asian populations in the allelic model and to oral cancer risk among the overall populations in the allelic model with oral cancer); it was weak for *MMP-2* rs243865 among the Asian populations with LC risk in the recessive model, among the overall populations with LC risk in the dominant model, and among the Asian population with NPC risk in the dominant model. *MMP-7* rs11568818 was related to CC risk among the Asian population in the recessive and dominant models, as well as with CRC risk among the Asian populations in the allelic and recessive models. *MMP-9* rs3918242 was associated with BC risk in all populations under the dominant model, GC risk in Asians under the recessive model, HCC risk in all populations under the recessive model, LC risk among the Caucasian and overall populations in the recessive and allelic models, and oral cancer risk in all populations under the allelic model (see **Table 1**).

### Heterogeneity, Bias, and Sensitivity Analyses

**Table 1** presents on heterogeneity, bias, and sensitivity analyses. There was low heterogeneity regarding the associations of *MMP-2* rs243865 (allelic model, *I* ^2^ = 0.0%, *p* = 0.837; dominant model, *I* ^2^ = 0.0%, *p* = 0.864; recessive model, *I* ^2^ = 0.9%, *p* = 0.944) in Asians with LC risk, *MMP-2* rs243865 (dominant model, *I* ^2^ = 7.1%, *p* = 0.371) in all populations with PCa risk, and *MMP-2* rs243865 (allelic model, *I* ^2^ = 0.0%, *p* = 0.405; dominant model, *I* ^2^ = 0.0%, *p* = 0.696) in Asians with PCa risk. Associations of *MMP-7* rs11568818 (allelic model, *I* ^2^ = 0.0%, *p* = 0.868; recessive model, *I* ^2^ = 0.0%, *p* = 0.962) were found in all populations and (allelic model, *I* ^2^ = 23.7%, *p* = 0.269; recessive model, *I* ^2^ = 0.0%, *p* = 0.494) in Asians with bladder cancer risk, *MMP-7* rs11568818 (allelic model, *I* ^2^ = 0.0%, *p* = 0.583) in Asians with CC risk and (allelic model, *I* ^2^ = 0.0%, *p* = 0.566; recessive model, *I* ^2^ = 12.6%, *p* = 0.285) in Asians with CRC risk. Furthermore, associations of *MMP-9* rs3918242 (dominant model, *I* ^2^ = 21.8%, *p* = 0.27; recessive model, *I* ^2^ = 0.0%, *p* = 0.723) were found in all populations and in Asians (allelic model, *I* ^2^ = 0.0%, *p* = 0.731; dominant model, *I* ^2^ = 0.0%, *p* = 0.628; recessive model, *I* ^2^ = 0.0%, *p* = 0.917) with BC risk (recessive model, *I* ^2^ = 0.0%, *p* = 0.551) in Asians with GC risk, in Caucasians (allelic model, *I* ^2^ = 0.0%, *p* = 0.502; recessive model, *I* ^2^ = 0.0%, *p* = 0.615) with LC risk, and in all populations (recessive model, *I* ^2^ = 0.0%, *p* = 0.717) with oral cancer risk; moderate heterogeneity was detected for relations of *MMP-2* rs243865 (allelic model, *I* ^2^ = 41.4%, *p* = 0.163; dominant model, *I* ^2^ = 46.9%, *p* = 0.13) in Asians with esophageal cancer risk and of *MMP-9* rs3918242 (allelic model, *I* ^2^ = 29.2%, *p* = 0.216) in all populations with BC risk (recessive model, *I* ^2^ = 48.1%, *p* = 0.165) and HCC risk and (recessive model, *I* ^2^ = 42.1%, *p* = 0.159) with LC risk. Furthermore, the relation of *MMP-9* rs3918242 (allelic model, *I* ^2^ = 26.8%, *p* = 0.255) in all populations with oral cancer risk was found; there was large heterogeneity regarding the associations of *MMP-2* rs243865 (allelic model, *I* ^2^ = 72.1%, *p* = 0.006; dominant model, *I* ^2^ = 73.8%, *p* = 0.004) in all populations with LC risk and in Asians (dominant model, *I* ^2^ = 63%, *p* = 0.067) with NPC risk, *MMP-7* rs11568818 (dominant model, *I* ^2^ = 52.8%, *p* = 0.12; recessive model, *I* ^2^ = 52.7%, *p* = 0.121) in Asians with CC risk, and *MMP-9* rs3918242 (allelic model, *I* ^2^ = 63.3%, *p* = 0.028) in all populations with LC risk. No significant publication bias was detected regarding the connections between *MMP* variants and cancer risk (*p* > 0.10), with the only exception of *MMP-2* rs243865 with LC risk among the overall populations in the dominant and allelic models. Sensitivity analysis was conducted for assessing the robustness of the significant associations. As a result, the summary ORs remained unchanged, despite deleting one single study, the first studies, or deviations from HWE among controls, with the only exception of *MMP-2* rs243865 with NPC risk among the Asian populations in dominant model, *MMP-7* rs11568818 with CC risk in Asians under the dominant model, and *MMP-9* rs3918242 with LC risk among the overall populations in the recessive model and with BC risk among the overall populations in the dominant model. In our sensitivity analyses, no significant correlation was observed for any of the three models, excluding works deviating from HWE among controls.

## Discussion

Although numerous studies have reported associations between *MMP-2*, *MMP-7*, and *MMP-9* variants and cancer risk, these results are highly controversial. Considering that, this study has the largest scale and is an integrative study that evaluates the relations of *MMP-2*, *MMP-7*, and *MMP-9* variants with cancer susceptibility. Relevant information was obtained in publications, and altogether 135 articles (36,530 cases and 41,258 controls) were collected for meta-analysis. In 2010, Peng et al. performed a meta-analysis involving 51 articles and over 40,000 participants (19) and found that *MMP-2*, *MMP-7*, and *MMP-9* variants are linked with the risk of cancer. Furthermore, compared with previous studies, our study included more studies and variants, and then it was revealed that *MMP-2* rs243865 was associated with NPC and PCa risk, and *MMP-7* rs11568818 with bladder cancer, CC, and CRC risk. Furthermore, *MMP-9* rs3918242 was related to BC, GC, HCC, LC, and oral cancer risk. Then, whether the cumulative epidemiological evidence regarding such obvious associations was creditable combined with the FPRP test and Venice criteria was assessed. At last, 12 associations (*MMP-2* rs243865 with esophageal cancer and LC, *MMP-7* rs11568818 with bladder and CC, and *MMP-9* rs3918242 with BC) were rated as strong evidence for cancer risk, 7 as moderate evidence, and 15 as weak.

Located on chromosome 16q21, *MMP-2* gene contains 13 exons and 12 introns (22), which mainly degrade gelatin and type IV collagen, the main structural components of BM, so it has been identified as a critical marker for cancer occurrence and migration (23). *MMP-2* binds to integrin αvβ3 through the hemopexin domain and is essential for mesenchymal cell invasion activity (24). In addition, rs243865 polymorphism of the *MMP-2* promoter can affect mRNA and protein expression by changing its transcriptional activity and can lead to the occurrence of some cancers (25–28). However, certain transcription factors (TFs), like specificity protein-1 (SP-1) and activator protein-1 (AP-1), have a direct influence on *MMP-2* transcription (18, 29). Furthermore, the SP-1 binding region is inactivated by rs243865, resulting in reduced transcription and translation of *MMP-2* (30). This work suggested that rs243865 was related to the risk of esophageal and LC under the allelic and dominant models, with 1.249-fold and 1.277-fold reduced incidence of esophageal cancer among the Asian populations in the dominant and allelic models, and 1.516-fold (with a sample size of 4254) and 1.466-fold (with a sample size of 2850) reduced LC risk in the dominant and allelic models in Asians, rather than under the recessive model in Asians with esophageal cancer and the recessive model in all populations with LC. Here, it was important to emphasize the associations between the risk of esophageal cancer with such single-nucleotide polymorphism (SNP) among the Asian populations in the allelic and dominant models, which were upgraded from moderate to strong (FPRP < 0.05). *MMP-2* has been previously found to show overexpression within various human cancers, such as ESCC and LC (15, 31–33). A high expression level of *MMP-2* is a potentially unfavorable factor that predicts tumorigenesis, but rs243865 leads to a lower expression of *MMP-2* with lower cancer risk. Furthermore, the finding of Price et al. in 2001 that C>T polymorphism, which was located at −1,306 and destroyed the SP-1 promoter site (CCACC box), showed remarkably decreased activity of *MMP-2* promoter relative to the C allele was further confirmed in our study (30). Nonetheless, in our study, only the Asian populations were analyzed, and we failed to analyze other ethnic groups such as Caucasians due to insufficient data or a small sample size. Therefore, large-scale research on other races in the future is recommended, which may show that biological characteristics of *MMP-2* rs243865 may have differences in different ethnic groups.

*MMP-7*, located on human chromosome 11q21–q22, represents a small secretory protease that shows wide substrate specificity, which is responsible for degrading proteoglycans, elastin, type IV collagen, and fibronectin (34, 35). It cleaves non-matrix substrates on the cell surface, such as Fas ligand, E-cadherin, and pro-cancer TNF-α, also referred to as the “sheddase” effect (36, 37). Its level is related to tumor migration, invasion, and prognosis. SNP 181A>G (rs11568818) is located in the *MMP-7* promoter region known to influence gene expression. Our meta-analysis strongly indicated that rs11568818 could increase the risk of bladder cancer in all populations with a sample of 2,377 under both the allelic and recessive models (OR = 1.204, 95% CI = 1.055–1.374; OR = 1.538, 95% CI = 1.198–1.974) and in Asians with a sample of 1,938 under the allelic model (OR = 1.229, 95% CI = 1.056–1.431), while it also increased the risk of CC in Asians under the allelic model (OR = 1.372, 95% CI = 1.148–1.640). In this case, it can be seen that our results are inconsistent with those of some previous studies (38), which may be related to sample size, environment, and living habits. Furthermore, it should be pointed out that our results are more reliable due to the larger sample size. Interestingly, we upgraded the associations (*MMP-7* rs11568818 and CC among the Asian populations in the allelic model and bladder cancer among the overall populations in the recessive model) from moderate to strong. The amount of evidence explains the mechanism of grading two associations “BAA” and “BAA” based on the Venice criteria; due to the FPRP value <0.05, the associations were rated as strong. Moreover, the lack of data from Caucasians in this study should be expanded and be recommended so as to further demonstrate this association in the future.

*MMP-9*, also called type IV collagenase or gelatinase B, is the protease degrading type IV collagen (the main BM component). It has a critical function in distant metastasis of tumor cells because of the lysis activity of type IV collagen that disrupts the BM (39). *MMP-9* promoter 1562C>T (rs3918242) functional polymorphism predicts a higher *MMP-9* expression level (40). Promoter activity increases by 1.5 times in the MMP-9 T allele in comparison with the *MMP-9* C allele (7). In this case, it is indicated that rs3918242 plays a very important role in the generation and metastasis of tumors, which is consistent with our results. To be specific, our meta-analysis strongly suggested that rs3918242 elevated the BC risk among the overall populations in the recessive and allelic models with 1.681-fold and 1.281-fold, respectively, and among the Asian populations in the recessive and dominant models with 1.710-fold and 1.526-fold, accordingly. However, this study sample lacked Caucasian population analysis. In other words, this work was featured with a small sample size, which was the cause of focusing on the overall population. More research regarding such SNP in different races should be recommended.

There were 7 associations graded as moderate associations for cancer risk, including *MMP-2* rs243865 with LC risk and PCa risk, *MMP-7* rs11568818 with bladder cancer risk, and *MMP-9* rs3918242 with BC risk and oral cancer risk. These 7 associations were rated as moderate evidence due to high heterogeneity, publication bias, and a small-study effect based on the Venice criteria and FPRP values. Furthermore, large prospective studies should be performed to elucidate the relationships between these variants with cancer risk.

There were 15 associations rated as weakly associated with cancer risk. Among these associations, *MMP-2* rs243865 was connected with LC risk and *MMP-9* rs3918242 with BC, HCC, LC, and oral cancer risk. They were all meaningful associations in all populations. Aside from that, other 7 associations were considered significant in Asians, including *MMP-2* rs243865 with LC risk and NPC risk, *MMP-7* rs11568818 with CC risk and CRC risk, and *MMP-9* rs3918242 with GC risk. However, 2 associations were regarded as significant in Caucasians, such as *MMP-9* rs3918242 with LC risk. In these variants, *MMP-2* rs243865 decreased the risk of LC by 1.387-fold under the dominant model in all populations with “ACC” based on the Venice criteria. Furthermore, a high degree of heterogeneity, a publication bias, or a small-study effect may explain why this variant was rated as weak evidence. Apart from that, *MMP-2* rs243865 decreased the risk of LC and the risk of NPC. Beyond that, *MMP-7* rs11568818 in Asians was associated with CRC risk with “BAA,” and the FPRP value >0.2 led from moderate grade to weak grade, which was mainly due to the low amount of evidence, high heterogeneity of the data, a publication bias, a small-study effect, and HWE bias on the Venice criteria. Moreover, expanding the sample size and evaluating additional race groups of such variants are important to further investigate these associations.

In addition, the association was inconsistent according to different ethnic or genetic models. In terms of ethnicity, except for the analysis on the association of *MMP-9* rs3918242 with LC risk among the Europeans, the other subgroup analyses on associations were mainly conducted in the Asian populations, whereas subgroup analysis was not made since insufficient non-Asians were enrolled. This study adopted three genetic models to comprehensively assess the associations; patients’ age, gender and other different genetic backgrounds, tumor subtypes, and environmental factors may be the variation source. More investigations into the above factors are necessary.

This study presented that three SNPs in two *MMP*s had no association with two cancers in any genetic model and/or ethnicity; of these, one SNP showed no relation with the risk of cancer (*MMP-2* rs243865 with BC) in meta-analyses that involved at least 2,000 cases and 2,000 controls, providing >85% power for detecting OR = 1.15 in the allelic model for the variant with type 1 error 0.05 and minor allelic frequency (MAF) 0.20 (**Supplementary Tables S3**, **S4**). Further research on this SNP with a similar sample size may not yield fruitful results. For the remaining SNPs, as these associations were characterized by low statistical power in the current sample size, further expanding the sample size or large meta-analyses on these associations are recommended.

Of course, in this study, there are some limitations: a) the literature collected in this study was in English, not in other languages, which may lead to bias; b) the subgroup analysis was performed only on Asians and Caucasians and under three genetic models, while other factors, such as age, gender, smoking, alcohol intake, and environment, were ignored, which might compromise our result reliability; c) only the susceptibility of associations between *MMP-2*, *MMP-7*, and *MMP-9* and cancer risk was assessed; furthermore, due to insufficient data, the influence of gene polymorphism on cancer progression and metastasis has not been evaluated. Regardless of the abovementioned limitations, the present work comprehensively investigated available publications to examine the functions of *MMP-2*, *MMP-7*, and *MMP-9* in cancers and will be valuable for future genetic studies.

The present work assessed cumulative epidemiological evidence supporting the obvious relations of *MMP*s with tumor susceptibility through integrating the FPRP test and Venice criteria. Finally, 12 associations (*MMP-2* rs243865 with esophageal cancer risk and LC risk, *MMP-7* rs11568818 with bladder risk and CC risk, and *MMP-9* rs3918242 with BC risk) were rated as strong evidence, 7 as moderate evidence, and 15 as weak. Analysis of the relations between *MMP*s variants and tumor susceptibility contributes to obtaining high-risk subjects for primary prevention. To sum up, this work reviews existing publications regarding *MMP* variations with tumor susceptibility. Our results offer valuable data to design future research to assess variants in *MMP* factors for cancer risk.

## Data Availability Statement

The original contributions presented in the study are included in the article/**Supplementary Material**. Further inquiries can be directed to the corresponding author.

## Author Contributions

CH and TW designed this work. CH and SX integrated and analyzed the data. CH and TW wrote this manuscript. CH, SX, ZL, DL, and RW finished the related tables and figures. CH and TW edited and revised the manuscript. All authors approved this manuscript.

## Conflict of Interest

The authors declare that the research was conducted in the absence of any commercial or financial relationships that could be construed as a potential conflict of interest.

## Publisher’s Note

All claims expressed in this article are solely those of the authors and do not necessarily represent those of their affiliated organizations, or those of the publisher, the editors and the reviewers. Any product that may be evaluated in this article, or claim that may be made by its manufacturer, is not guaranteed or endorsed by the publisher.
