# Supplementary material for: Epidemiological Evidence Between Variants in Matrix Metalloproteinases-2, -7, and -9 and Cancer Risk
Source: Front Oncol. 2022 Apr 28;12:856831. doi: 10.3389/fonc.2022.856831 (PMC9095957; doi:10.3389/fonc.2022.856831)
Supplement: Supplementary file 1 [file Image_1.pdf]

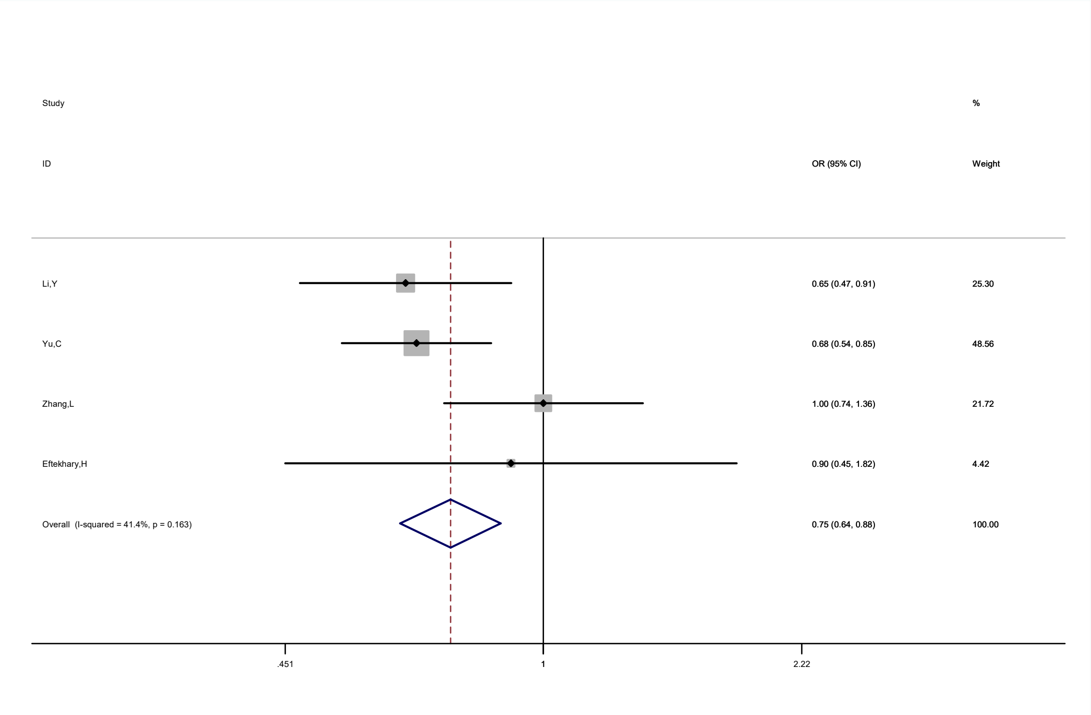


Supplementary Figure S1. The association between *MMP-2* rs243865 and risk of esophageal cancer in Asians under allelic model.

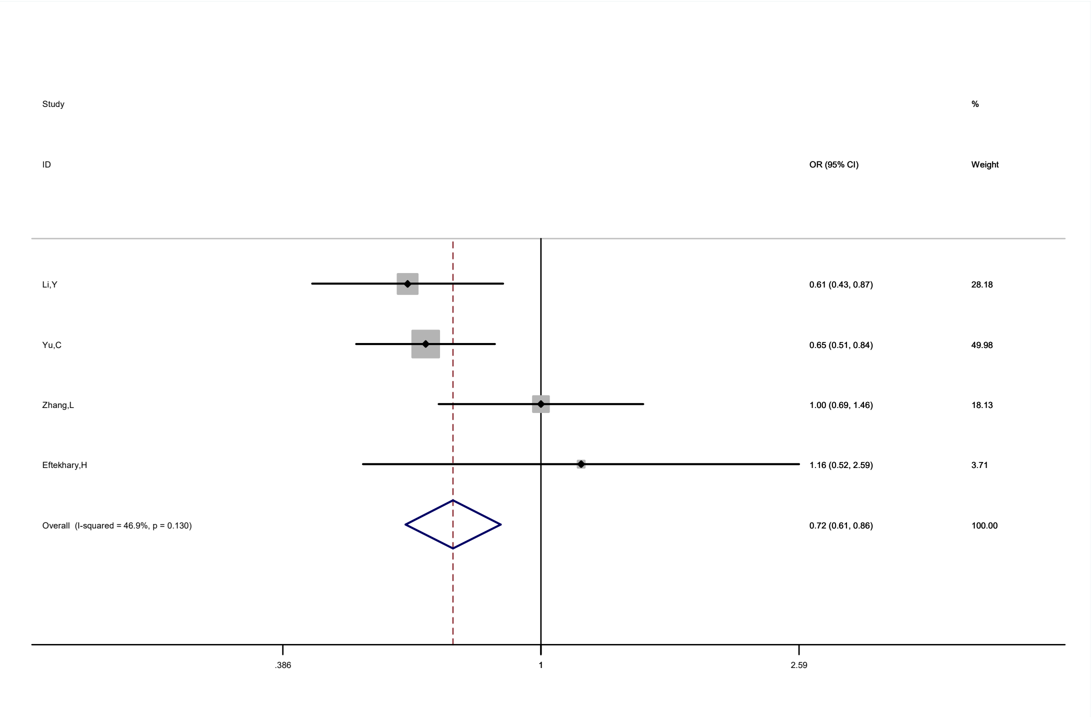


Supplementary Figure S2. The association between *MMP-2* rs243865 and risk of esophageal cancer in Asians under dominant model.

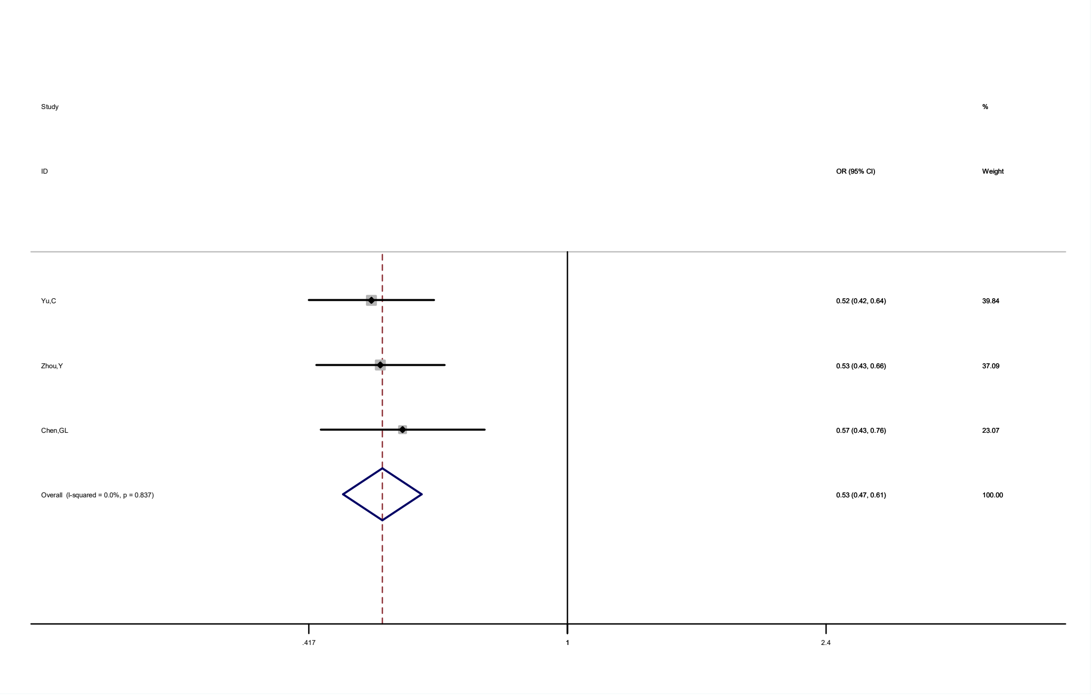


Supplementary Figure S3. The association between *MMP-2* rs243865 and risk of lung cancer in Asians under allelic model.

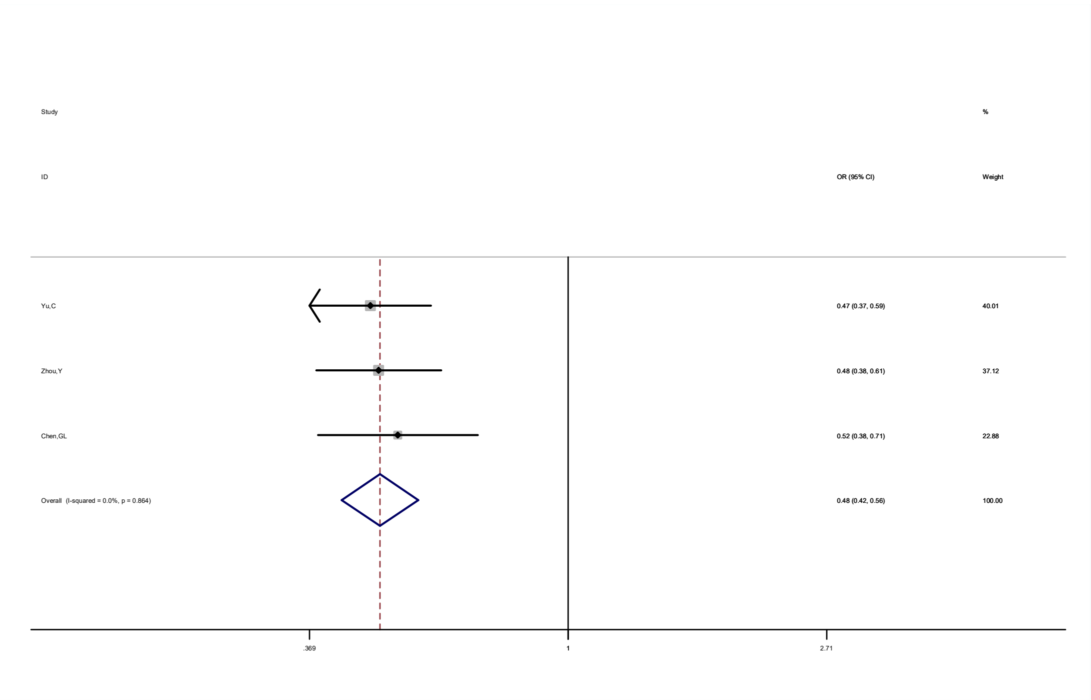


Supplementary Figure S4. The association between *MMP-2* rs243865 and risk of lung cancer in Asians under dominant model.

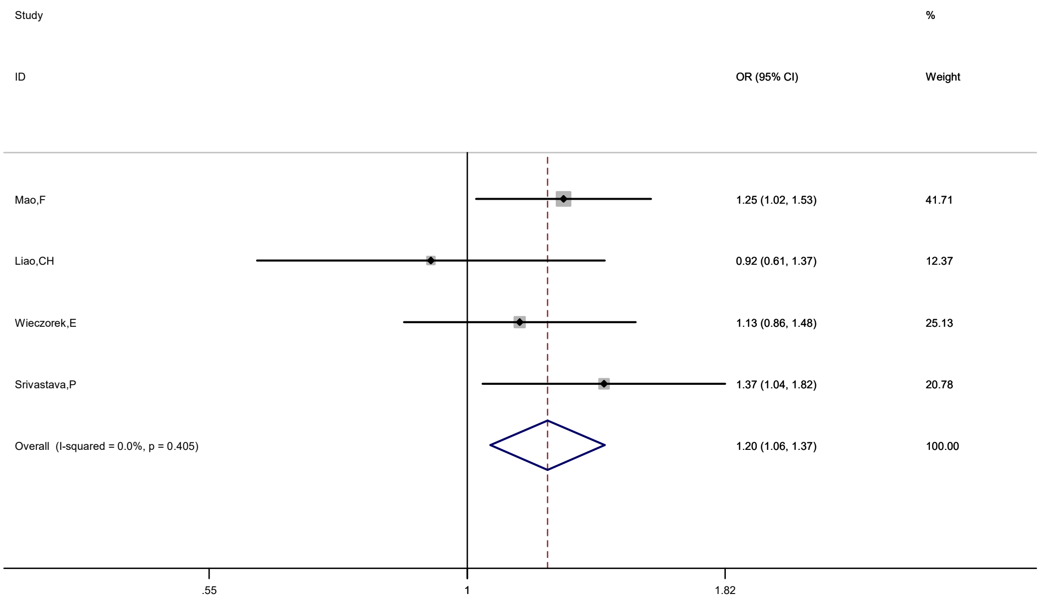


Supplementary Figure S5. The association between *MMP-7* rs11568818 and risk of bladder cancer in all populations under allelic model.

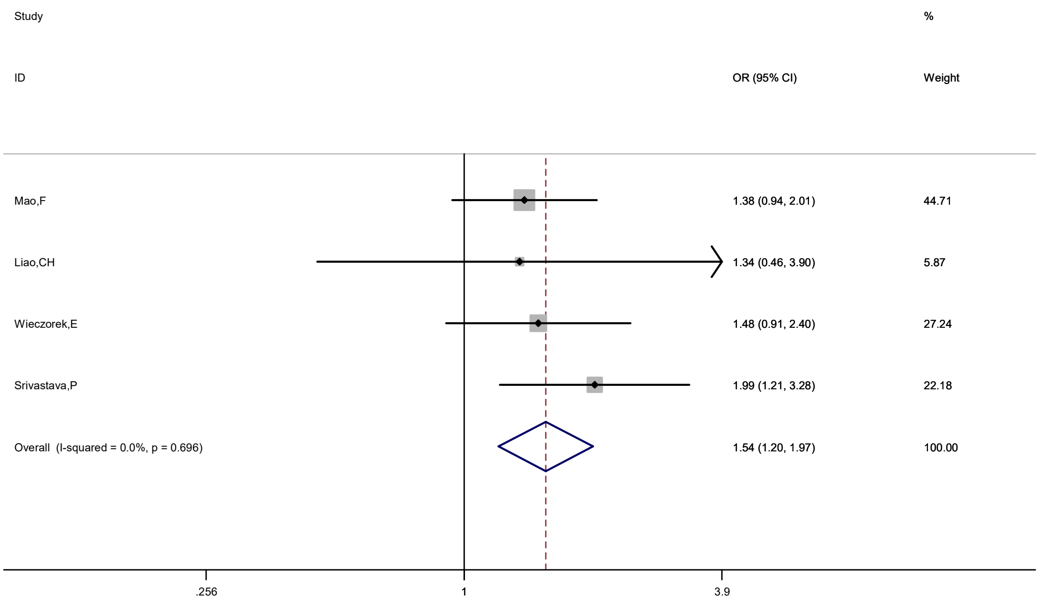


Supplementary Figure S6. The association between *MMP-7* rs11568818 and risk of bladder cancer in all populations under recessive model.

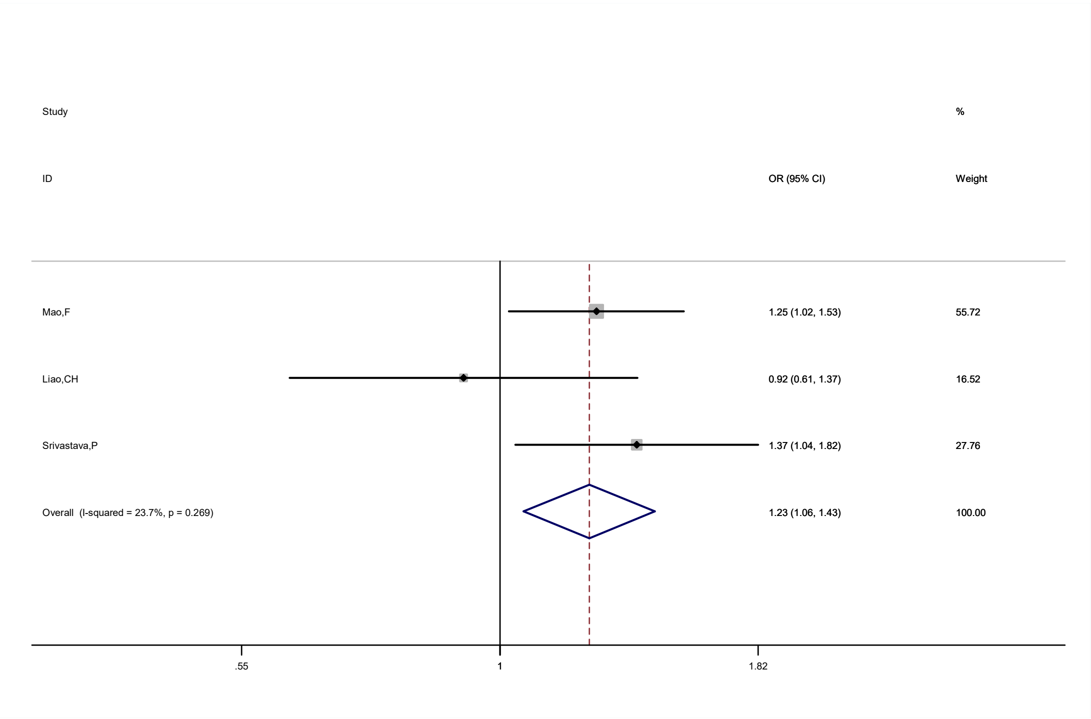


Supplementary Figure S7.The association between *MMP-7* rs11568818 and risk of bladder cancer in Asians under allelic model.

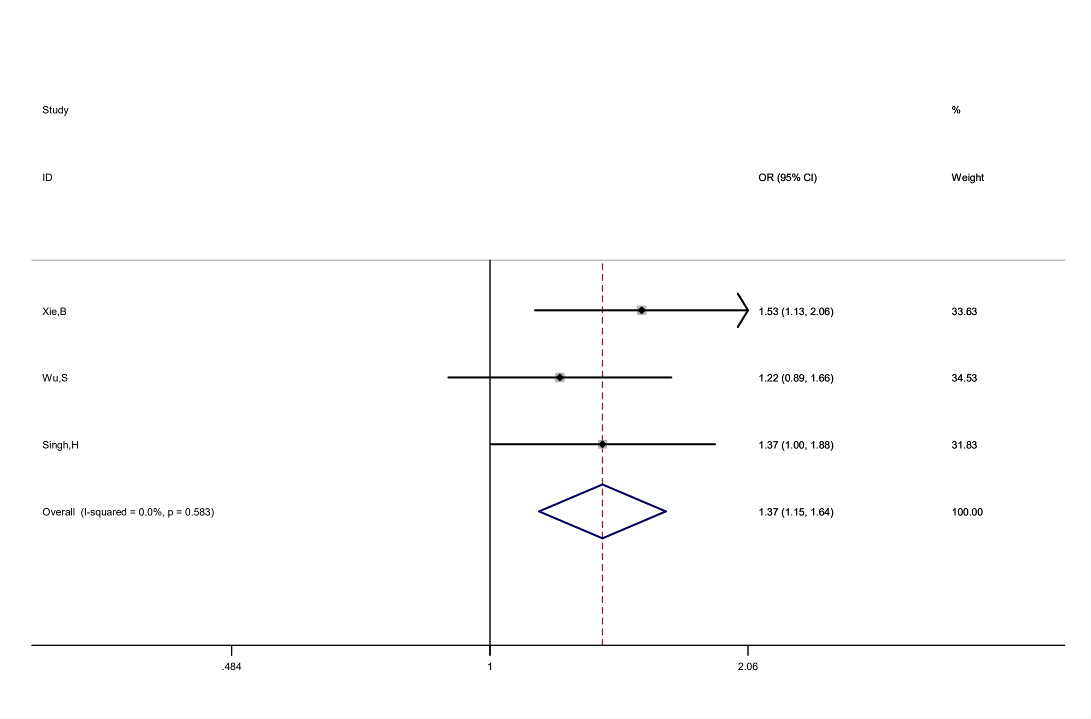


Supplementary Figure S8. The association between *MMP-7* rs11568818 and risk of cervical cancer in Asians under allelic model.

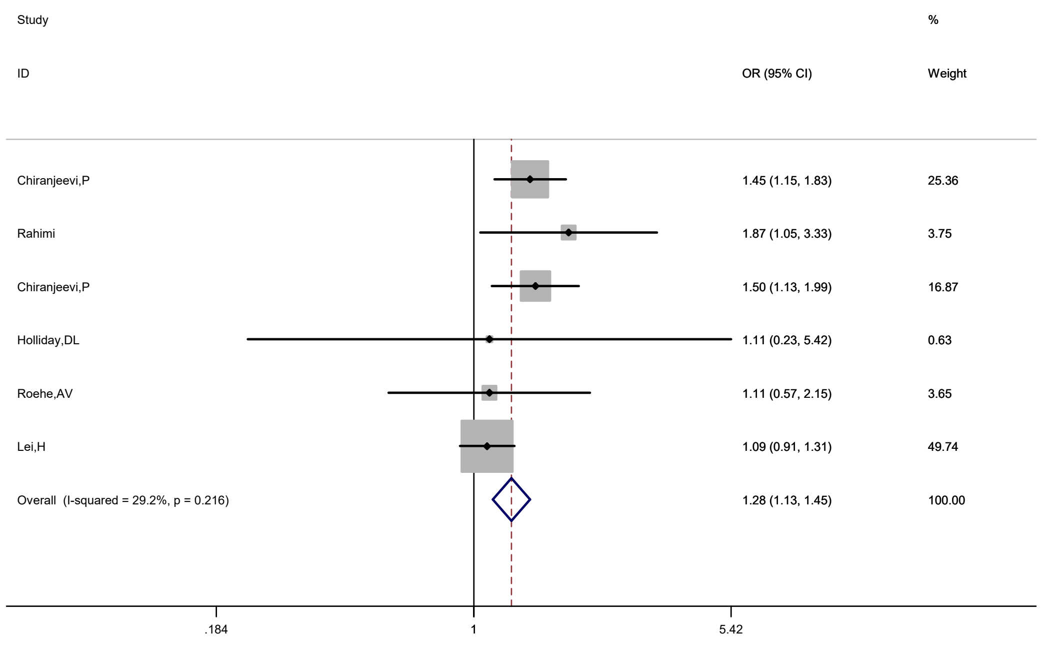


Supplementary Figure S9. The association between *MMP-9* rs3918242 and risk of breast cancer in all populations under allelic model.

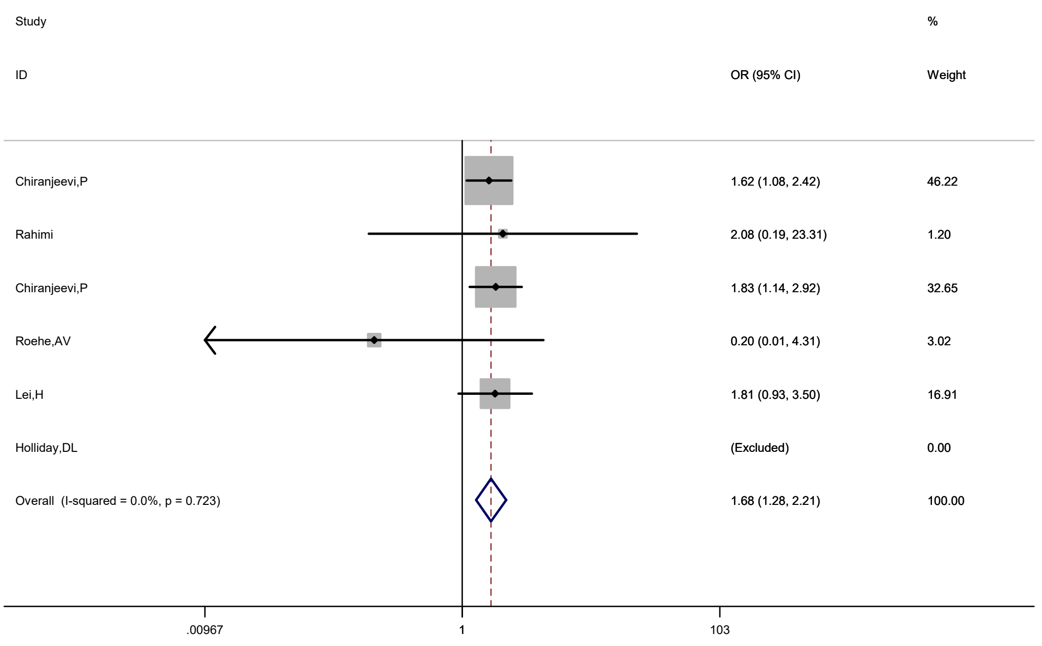


Supplementary Figure S10. The association between *MMP-9* rs3918242 and risk of breast cancer in all populations under recessive model.

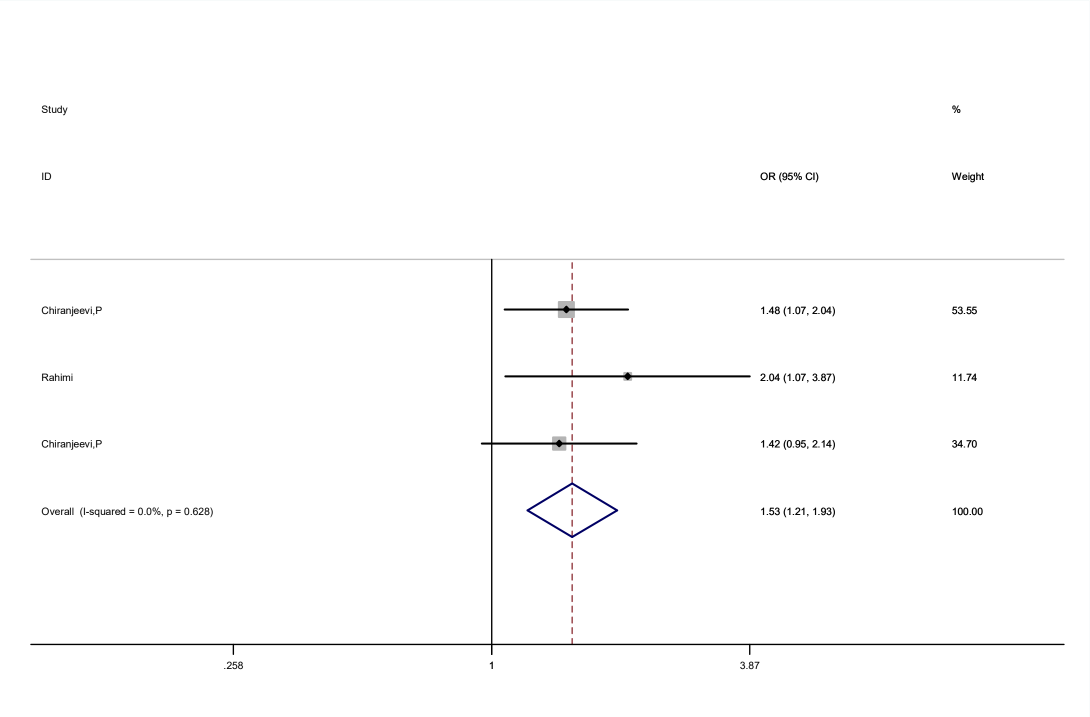


Supplementary Figure S11. The association between *MMP-9* rs3918242 and risk of breast cancer in Asians under dominant model.

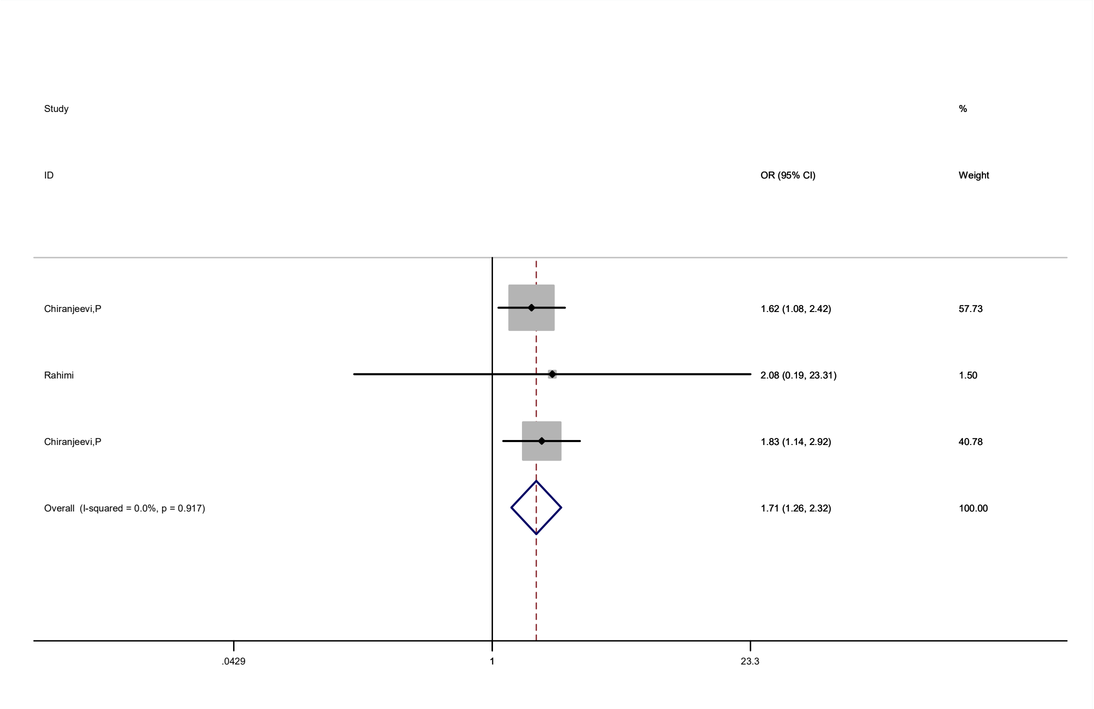


Supplementary Figure S12. The association between *MMP-9* rs3918242 and risk of breast cancer in Asians under recessive model.
