# Supplementary material for: Epidemiological Evidence Between Variants in Matrix Metalloproteinases-2, -7, and -9 and Cancer Risk
Source: Front Oncol. 2022 Apr 28;12:856831. doi: 10.3389/fonc.2022.856831 (PMC9095957; doi:10.3389/fonc.2022.856831)
Supplement: Supplementary file 3 [file Table_2.docx]

Supplementary Table S2： Characteristics of the included articles

| **No.** | **First author** | **Publishing Year** | **Case** | **Control** | **Cancer site** | **Country/region** | | **Ethnicity** | **Study design** | **MMP** | **rs number** | **Allelic** |
| --- | --- | --- | --- | --- | --- | --- | --- | --- | --- | --- | --- | --- |
| 1 | Miao, X | 2003 | 356 | 789 | Gastric | China | | Asian | CCS | MMP-2 | rs243865 | TvsC |
| 2 | Wu, CY | 2007 | 240 | 283 | Gastric | China | | Asian | CCS | MMP-2 | rs243865 | TvsC |
| 3 | Sugimoto, M | 2008 | 160 | 156 | Gastric | Japan | | Asian | CCS | MMP-7 | rs11568818 | GvsA |
| 4 | Liu,L | 2011 | 344 | 324 | Gastric | China | | Asian | CCS | MMP-2 | rs243865 | TvsC |
| 5 | Malik,MA | 2011 | 108 | 195 | Gastric | India | | Asian | CCS | MMP-7 | rs11568818 | GvsA |
| 6 | JiHye,K | 2011 | 153 | 326 | Gastric | Korea | | Asian | CCS | MMP-2 | rs243865 | TvsC |
|  | JiHye,K | 2011 | 153 | 326 | Gastric | Korea | | Asian | CCS | MMP-7 | rs11568818 | GvsA |
| 7 | Kesh,K | 2015 | 260 | 260 | Gastric | India | | Asian | CCS | MMP-7 | rs11568818 | CvsT |
| 8 | Avcı,N | 2015 | 79 | 65 | Gastric | Turkey | | Caucasian | CCS | MMP-9 | rs3918242 | TvsC |
| 9 | Krishnaveni,D | 2012 | 140 | 132 | Gastric | India | | Asian | CCS | MMP-9 | rs3918242 | TvsC |
| 10 | Zhang,DY | 2015 | 254 | 250 | Gastric | China | | Asian | CCS | MMP-2 | rs243865 | TvsC |
|  | Zhang DY | 2015 | 254 | 250 | Gastric | China | | Asian | CCS | MMP-2 | rs1053605 | TvsC |
| 11 | Fu,CK | 2020 | 121 | 363 | Gastric | China | | Asian | CCS | MMP-7 | rs11568818 | GvsA |
|  | Fu,CK | 2020 | 121 | 363 | Gastric | China | | Asian | CCS | MMP-7 | rs11568819 | TvsC |
| 12 | Li,Y | 2009 | 257 | 630 | Gastric | China | | Asian | CCS | MMP-2 | rs243865 | TvsC |
|  | Li,Y | 2009 | 335 | 624 | Esophageal | China | | Asian | CCS | MMP-2 | rs243865 | TvsC |
|  | Li,Y | 2009 | 257 | 624 | Gastric | China | | Asian | CCS | MMP-2 | rs1053605 | TvsC |
|  | Li,Y | 2009 | 335 | 624 | Esophageal | China | | Asian | CCS | MMP-2 | rs1053605 | TvsC |
| 13 | Yu C | 2004 | 527 | 777 | Esophageal | China | | Asian | CCS | MMP-2 | rs243865 | TvsC |
|  | Yu C | 2004 | 527 | 777 | Esophageal | China | | Asian | CCS | MMP-2 | rs1053605 | TvsC |
| 14 | Wu J D | 2008 | 132 | 132 | Esophageal | China | | Asian | CCS | MMP-9 | rs17576 | GvsA |
|  | Wu J D | 2008 | 132 | 132 | Esophageal | China | | Asian | CCS | MMP-9 | rs2250889 | CvsG |
|  | Wu J D | 2008 | 132 | 132 | Esophageal | China | | Asian | CCS | MMP-9 | rs17577 | AvsG |
| 15 | Malik MA | 2011 | 135 | 195 | Esophageal | India | | Asian | CCS | MMP-7 | rs11568818 | GvsA |
| 16 | Guan,X | 2014 | 132 | 132 | Esophageal | China | | Asian | CCS | MMP-9 | rs3918242 | TvsC |
| 17 | Zhang L | 2015 | 226 | 226 | Esophageal | China | | Asian | CCS | MMP-2 | rs243865 | TvsC |
|  | Zhang,L | 2015 | 226 | 226 | Esophageal | China | | Asian | CCS | MMP-9 | rs3918242 | TvsC |
| 18 | Eftekhary H | 2015 | 70 | 60 | Esophageal | Iran | | Asian | CCS | MMP-2 | rs243865 | TvsC |
|  | Eftekhary,H | 2015 | 70 | 60 | Esophageal | Iran | | Asian | CCS | MMP-9 | rs3918242 | TvsC |
| 19 | Wang N | 2020 | 505 | 510 | Colorectal | China | | Asian | CCS | MMP-2 | rs1053605 | TvsC |
| 20 | Wu,MH | 2019 | 362 | 362 | Colorectal | China | | Asian | CCS | MMP-9 | rs3918242 | TvsC |
| 21 | Yueh,TC | 2018 | 362 | 362 | Colorectal | China | | Asian | CCS | MMP-7 | rs11568818 | CvsT |
|  | Yueh TC | 2018 | 362 | 362 | Colorectal | China | | Asian | CCS | MMP-7 | rs11568819 | TvsC |
| 22 | Ben Néjima D | 2017 | 116 | 150 | Colorectal | France | | Caucasian | CCS | MMP-2 | rs243865 | TvsC |
| 23 | Banday MZ | 2016 | 142 | 184 | Colorectal | India | | Asian | CCS | MMP-2 | rs243865 | TvsC |
|  | Banday,MZ | 2016 | 142 | 184 | Colorectal | India | | Asian | CCS | MMP-7 | rs11568818 | CvsT |
|  | Banday,MZ | 2016 | 142 | 184 | Colorectal | India | | Asian | CCS | MMP-9 | rs3918242 | TvsC |
| 24 | Moreno-Ortiz,JM | 2014 | 102 | 125 | Colorectal | Mexico | | Latin American | CCS | MMP-7 | rs11568818 | CvsT |
| 25 | Saeed HM | 2013 | 95 | 122 | Colorectal | Egypt | | African | CCS | MMP-2 | rs243865 | TvsC |
| 26 | Yang ZH | 2012 | 344 | 386 | Colorectal | China | | Asian | CCS | MMP-9 | rs2250889 | CvsG |
|  | Yang ZH | 2012 | 344 | 386 | Colorectal | China | | Asian | CCS | MMP-9 | rs2274756 | AvsG |
| 27 | Dziki,L | 2011 | 184 | 205 | Colorectal | Poland | | Caucasian | CCS | MMP-7 | rs11568818 | CvsT |
| 28 | Kang MJ | 2011 | 50 | 50 | Colorectal | Korea | | Asian | CCS | MMP-2 | rs243865 | TvsC |
| 29 | Park KS | 2011 | 333 | 318 | Colorectal | Korea | | Asian | CCS | MMP-2 | rs1053605 | TvsC |
| 30 | Fang,WL | 2011 | 237 | 252 | Colorectal | China | | Asian | CCS | MMP-7 | rs11568818 | CvsT |
|  | Fang,WL | 2011 | 237 | 252 | Colorectal | China | | Asian | CCS | MMP-9 | rs17576 | GvsA |
| 31 | de Lima,JM | 2009 | 130 | 130 | Colorectal | Brazil | | Mixed | CCS | MMP-7 | rs11568818 | CvsT |
| 32 | Xing,LL | 2007 | 137 | 199 | Colorectal | China | | Asian | CCS | MMP-9 | rs3918242 | TvsC |
|  | Xing,LL | 2007 | 137 | 199 | Colorectal | China | | Asian | CCS | MMP-9 | rs17576 | GvsA |
| 33 | Woo,M | 2007 | 185 | 304 | Colorectal | Korea | | Asian | CCS | MMP-7 | rs11568818 | CvsT |
|  | Woo,M | 2007 | 185 | 304 | Colorectal | Korea | | Asian | CCS | MMP-9 | rs3918242 | TvsC |
|  | Woo,M | 2007 | 185 | 304 | Colorectal | Korea | | Asian | CCS | MMP-9 | rs17576 | GvsA |
| 34 | Lièvre,A | 2006 | 597 | 568 | Colorectal | France | | Caucasian | CCS | MMP-7 | rs11568818 | CvsT |
| 35 | Elander N | 2006 | 127 | 208 | Colorectal | Sweden | | Caucasian | CCS | MMP-2 | rs243865 | TvsC |
|  | Elander,N | 2006 | 127 | 208 | Colorectal | Sweden | | Caucasian | CCS | MMP-9 | rs3918242 | TvsC |
| 36 | Xu E | 2004 | 126 | 126 | Colorectal | China | | Asian | CCS | MMP-2 | rs243865 | TvsC |
| 37 | Samanoudy,A | 2014 | 131 | 60 | Hepatocellular | Egypt | | African | CCS | MMP-9 | rs3918242 | TvsC |
| 38 | Qiu W D | 2008 | 434 | 480 | Hepatocellular | China | | Asian | CCS | MMP-7 | rs11568818 | GvsA |
| 39 | Sharma KL | 2012 | 410 | 230 | Gallbladder | India | | Asian | CCS | MMP-2 | rs1053605 | TvsC |
|  | Sharma KL | 2012 | 410 | 230 | Gallbladder | India | | Asian | CCS | MMP-2 | rs243865 | TvsC |
|  | Sharma KL | 2012 | 410 | 230 | Gallbladder | India | | Asian | CCS | MMP-7 | rs11568818 | GvsA |
|  | Sharma KL | 2012 | 410 | 230 | Gallbladder | India | | Asian | CCS | MMP-9 | rs17556 | QvsR |
|  | Sharma KL | 2012 | 410 | 230 | Gallbladder | India | | Asian | CCS | MMP-9 | rs2250889 | CvsG |
|  | Sharma KL | 2012 | 410 | 230 | Gallbladder | India | | Asian | CCS | MMP-9 | rs17577 | AvsG |
| 40 | Hsu,SW | 2019 | 208 | 416 | Nasopharyngeal | China | | Asian | CCS | MMP-2 | rs243865 | TvsC |
| 41 | Shao,JY | 2012 | 370 | 390 | Nasopharyngeal | China | | Asian | CCS | MMP-2 | rs243865 | TvsC |
| 42 | Zhou,G | 2007 | 832 | 766 | Nasopharyngeal | China | | Asian | CCS | MMP-2 | rs243865 | TvsC |
|  | Zhou G | 2007 | 832 | 766 | Nasopharyngeal | China | | Asian | CCS | MMP-2 | rs1053605 | TvsC |
|  | Zhou G | 2007 | 832 | 766 | Nasopharyngeal | China | | Asian | CCS | MMP-7 | rs11568818 | GvsA |
|  | Zhou G | 2007 | 832 | 766 | Nasopharyngeal | China | | Asian | CCS | MMP-9 | rs3918242 | TvsC |
| 43 | Nasr HB | 2007 | 174 | 171 | Nasopharyngeal | Tunisia | | African | CCS | MMP-9 | rs3918242 | TvsC |
| 44 | Vairaktaris E | 2008 | 162 | 168 | Oral | | Greece+Germany | Caucasian | CCS | MMP-9 | rs3918242 | TvsC |
| 45 | Tsai,CW | 2018 | 788 | 956 | Oral | China | | Asian | CCS | MMP-2 | rs243865 | TvsC |
|  | Tsai CW | 2018 | 788 | 956 | Oral | China | | Asian | CCS | MMP-2 | rs1053605 | TvsC |
| 46 | Shih LC | 2018 | 788 | 956 | Oral | China | | Asian | CCS | MMP-7 | rs11568818 | GvsA |
|  | Shih LC | 2018 | 788 | 956 | Oral | China | | Asian | CCS | MMP-7 | rs11568819 | TvsC |
| 47 | Yete,S | 2017 | 500 | 500 | Oral | India | | Asian | CCS | MMP-2 | rs243865 | TvsC |
|  | Yete S | 2017 | 500 | 500 | Oral | India | | Asian | CCS | MMP-2 | rs243842 | CvsT |
| 48 | Chaudhary,AK | 2011 | 426 | 422 | Oral | India | | Asian | CCS | MMP-2 | rs243865 | TvsC |
|  | Chaudhary AK | 2011 | 426 | 422 | Oral | India | | Asian | CCS | MMP-9 | rs3918242 | TvsC |
| 49 | Vairaktaris E | 2007 | 159 | 120 | Oral | Greek+German | | Caucasian | CCS | MMP-7 | rs11568818 | GvsA |
| 50 | Tu HF | 2007 | 192 | 191 | Oral | China | | Asian | CCS | MMP-9 | rs3918242 | TvsC |
| 51 | Lin,SC | 2004 | 121 | 147 | Oral | China | | Asian | CCS | MMP-2 | rs243865 | TvsC |
| 52 | Zhang H | 2017 | 150 | 120 | Cervical | China | | Asian | CCS | MMP-2 | rs1053605 | TvsC |
|  | Zhang H | 2017 | 150 | 120 | Cervical | China | | Asian | CCS | MMP-9 | rs3918242 | TvsC |
| 53 | Singh N | 2016 | 150 | 150 | Cervical | India | | Asian | CCS | MMP-2 | rs243865 | TvsC |
| 54 | Xie B | 2016 | 230 | 230 | Cervical | China | | Asian | CCS | MMP-2 | rs243865 | TvsC |
|  | Xie,B | 2016 | 230 | 230 | Cervical | China | | Asian | CCS | MMP-7 | rs11568818 | GvsA |
|  | Xie B | 2016 | 230 | 230 | Cervical | China | | Asian | CCS | MMP-9 | rs3918242 | TvsC |
| 55 | Srivastava P | 2013 | 200 | 200 | Cervical | India | | Asian | CCS | MMP-2 | rs1053605 | TvsC |
| 56 | Wu,S | 2011 | 217 | 190 | Cervical | China | | Asian | CCS | MMP-7 | rs11568818 | GvsA |
| 57 | Singh,H | 2008 | 150 | 162 | Cervical | India | | Asian | CCS | MMP-7 | rs11568818 | GvsA |
| 58 | Baltazar-Rodriguez LM | 2008 | 54 | 126 | Cervical | Mexico | | Latin American | CCS | MMP-2 | rs243865 | TvsC |
| 59 | Yu-Chiao Yi | 2010 | 118 | 229 | Endometrial | China | | Asian | CCS | MMP-2 | rs1053605 | TvsC |
|  | Yu-Chiao Yi | 2010 | 118 | 229 | Endometrial | China | | Asian | CCS | MMP-7 | rs11568818 | GvsA |
| 60 | Wang Y | 2015 | 417 | 417 | Ovarian | America | | Caucasian | CCS | MMP-9 | rs6094237 | AvsT |
| 61 | Li Y | 2006 | 138 | 160 | Ovarian | China | | Asian | CCS | MMP-7 | rs11568818 | GvsA |
|  | Li Y | 2006 | 138 | 160 | Ovarian | China | | Asian | CCS | MMP-9 | rs3918242 | TvsC |
| 62 | Roncevic J | 2019 | 105 | 43 | Thyroid | Serbia | | Caucasian | CCS | MMP-9 | rs3918242 | TvsC |
| 63 | Nan H | 2008 | 803 | 870 | Skin | America | | Caucasian | CCS | MMP-9 | rs1805088 | TvsC |
|  | Nan H | 2008 | 803 | 870 | Skin | America | | Caucasian | CCS | MMP-9 | rs17576 | GvsA |
|  | Nan H | 2008 | 803 | 870 | Skin | America | | Caucasian | CCS | MMP-9 | rs2250889 | CvsG |
|  | Nan H | 2008 | 803 | 870 | Skin | America | | Caucasian | CCS | MMP-9 | rs2274756 | GvsA |
| 64 | Radunovic M | 2016 | 93 | 100 | Salivary gland | Serbia | | Caucasian | CCS | MMP-2 | rs243865 | TvsC |
|  | Radunovic M | 2016 | 93 | 100 | Salivary gland | Serbia | | Caucasian | CCS | MMP-2 | rs243866 | AvsG |
|  | Radunovic M | 2016 | 93 | 100 | Salivary gland | Serbia | | Caucasian | CCS | MMP-9 | rs3918242 | TvsC |
| 65 | Strbac D | 2018 | 236 | 161 | Malignant pleural | Slovenia | | Caucasian | CCS | MMP-2 | rs243865 | TvsC |
|  | Strbac D | 2018 | 236 | 161 | Malignant pleural | Slovenia | | Caucasian | CCS | MMP-2 | rs243849 | CvsT |
|  | Strbac D | 2018 | 236 | 161 | Malignant pleural | Slovenia | | Caucasian | CCS | MMP-2 | rs7201 | CvsA |
|  | Strbac D | 2018 | 236 | 161 | Malignant pleural | Slovenia | | Caucasian | CCS | MMP-9 | rs17576 | GvsA |
|  | Strbac D | 2018 | 236 | 161 | Malignant pleural | Slovenia | | Caucasian | CCS | MMP-9 | rs2250889 | CvsG |
|  | Strbac D | 2018 | 236 | 161 | Malignant pleural | Slovenia | | Caucasian | CCS | MMP-9 | rs17577 | AvsG |
|  | Strbac D | 2018 | 236 | 161 | Malignant pleural | Slovenia | | Caucasian | CCS | MMP-9 | rs20544 | TvsC |
| 66 | Lin,CM | 2017 | 376 | 352 | Lymphoma | China | | Asian | CCS | MMP-2 | rs243865 | TvsC |
|  | Lin,CM | 2017 | 376 | 352 | Lymphoma | China | | Asian | CCS | MMP-9 | rs3918242 | TvsC |
| 67 | Gouda HM | 2014 | 100 | 100 | Lymphoma | Egypt | | African | CCS | MMP-2 | rs1053605 | TvsC |
|  | Gouda,HM | 2014 | 100 | 100 | Lymphoma | Egypt | | African | CCS | MMP-2 | rs243865 | TvsC |
| 68 | Vasku A | 2010 | 89 | 198 | Lymphoma | Czech Republic | | Caucasian | CCS | MMP-2 | rs243866 | AvsG |
|  | Vasku,A | 2010 | 89 | 198 | Lymphoma | Czech Republic | | Caucasian | CCS | MMP-2 | rs243865 | TvsC |
|  | Vasku A | 2010 | 89 | 198 | Lymphoma | Czech Republic | | Caucasian | CCS | MMP-2 | rs243864 | GvsT |
| 69 | Liutkevicius V | 2020 | 112 | 458 | Laryngeal | Lithuania | | Caucasian | CCS | MMP-2 | rs1053605 | TvsC |
|  | Liutkevicius V | 2020 | 112 | 458 | Laryngeal | Lithuania | | Caucasian | CCS | MMP-2 | rs243865 | TvsC |
|  | Liutkevicius V | 2020 | 112 | 458 | Laryngeal | Lithuania | | Caucasian | CCS | MMP-9 | rs3918242 | TvsC |
| 70 | [O-charoenrat P](https://www.ncbi.nlm.nih.gov/pubmed/?term=O-charoenrat%20P%5bAuthor%5d&cauthor=true&cauthor_uid=16353148" \o "https://www.ncbi.nlm.nih.gov/pubmed/?term=O-charoenrat P[Author]&cauthor=true&cauthor_uid=16353148) | 2006 | 239 | 250 | Head and Neck | Thailand | | Asian | CCS | MMP-2 | rs243865 | TvsC |
| 71 | Ozden M | 2017 | 100 | 100 | Glioblastoma | Turkey | | Caucasian | CCS | MMP-9 | rs17576 | GvsA |
|  | Ozden M | 2017 | 100 | 100 | Glioblastoma | Turkey | | Caucasian | CCS | MMP-9 | rs2250889 | CvsG |
|  | Ozden M | 2017 | 100 | 100 | Glioblastoma | Turkey | | Caucasian | CCS | MMP-9 | rs17577 | AvsG |
| 72 | Kawal P | 2016 | 30 | 30 | Glioblastoma | India | | Asian | CCS | MMP-2 | rs243865 | TvsC |
|  | Kawal P | 2016 | 30 | 30 | Glioblastoma | India | | Asian | CCS | MMP-7 | rs11568818 | GvsA |
| 73 | Raj Kumar | 2011 | 110 | 150 | Glioblastoma | India | | Asian | CCS | MMP-2 | rs243865 | TvsC |
| 74 | Lu Z | 2007 | 236 | 366 | Glioblastoma | China | | Asian | CCS | MMP-9 | rs3918242 | TvsC |
| 75 | Lu Z | 2006 | 221 | 366 | Glioblastoma | China | | Asian | CCS | MMP-7 | rs11568818 | GvsA |
| 76 | Pei JS | 2017 | 266 | 266 | Leukemia | China | | Asian | CCS | MMP-7 | rs11568818 | GvsA |
|  | Pei JS | 2017 | 266 | 266 | Leukemia | China | | Asian | CCS | MMP-7 | rs11568819 | TvsC |
| 77 | Pirooz,HJ | 2018 | 100 | 100 | Gastric | Iran | | Asian | CCS | MMP-9 | rs1056628 | CvsA |
|  | Pirooz,HJ | 2018 | 100 | 100 | Breast | Iran | | Asian | CCS | MMP-9 | rs1056628 | CvsA |
|  | Pirooz,HJ | 2018 | 100 | 100 | Lung | Iran | | Asian | CCS | MMP-9 | rs1056628 | CvsA |
| 78 | Zhang J | 2005 | 258 | 350 | Esophageal | China | | Asian | CCS | MMP-7 | rs11568818 | GvsA |
|  | Zhang,J | 2005 | 201 | 350 | Gastric | China | | Asian | CCS | MMP-7 | rs11568818 | GvsA |
|  | Zhang,J | 2005 | 243 | 350 | Lung | China | | Asian | CCS | MMP-7 | rs11568818 | GvsA |
| 79 | Liao CH | 2017 | 92 | 580 | Renal | China | | Asian | CCS | MMP-7 | rs11568818 | GvsA |
|  | Liao CH | 2017 | 92 | 580 | Renal | China | | Asian | CCS | MMP-7 | rs11568819 | TvsC |
| 80 | Awakura Y | 2006 | 179 | 211 | Renal | Japan | | Asian | CCS | MMP-9 | rs3918242 | TvsC |
|  | Awakura Y | 2006 | 179 | 211 | Renal | Japan | | Asian | CCS | MMP-9 | rs17576 | GvsA |
| 81 | Białkowska,K | 2018 | 197 | 197 | Prostate | poland | | Caucasian | CCS | MMP-2 | rs243865 | TvsC |
|  | Białkowska K | 2018 | 197 | 197 | Prostate | poland | | Caucasian | CCS | MMP-7 | rs11568818 | GvsA |
| 82 | Shajarehpoor,SL | 2017 | 50 | 54 | Prostate | Iran | | Asian | CCS | MMP-2 | rs243865 | TvsC |
| 83 | Adabi,Z | 2015 | 102 | 139 | Prostate | Iran | | Asian | CCS | MMP-2 | rs243865 | TvsC |
| 84 | Yaykasli,KO | 2014 | 61 | 46 | Prostate | Turkey | | Caucasian | CCS | MMP-2 | rs243865 | TvsC |
| 85 | Srivastava,P | 2012 | 190 | 200 | Prostate | Indian | | Asian | CCS | MMP-2 | rs243865 | TvsC |
|  | Srivastava P | 2012 | 190 | 200 | Prostate | Indian | | Asian | CCS | MMP-2 | rs1053605 | TvsC |
| 86 | DosReis,ST | 2009 | 100 | 100 | Prostate | Brazil | | Mixed | CCS | MMP-2 | rs243865 | TvsC |
|  | Dos Reis ST | 2009 | 100 | 100 | Prostate | Brazil | | Mixed | CCS | MMP-7 | rs11568818 | GvsA |
|  | Dos Reis ST | 2009 | 100 | 100 | Prostate | Brazil | | Mixed | CCS | MMP-9 | rs17576 | GvsA |
| 87 | Sfar S | 2007 | 101 | 106 | Prostate | Tunisia | | African | CCS | MMP-9 | rs3918242 | TvsC |
| 88 | Mao,F | 2019 | 355 | 435 | Bladder | China | | Asian | CCS | MMP-7 | rs11568818 | GvsA |
| 89 | Liao,CH | 2018 | 375 | 375 | Bladder | China | | Asian | CCS | MMP-7 | rs11568818 | GvsA |
|  | Liao CH | 2018 | 375 | 375 | Bladder | China | | Asian | CCS | MMP-7 | rs11568819 | TvsC |
| 90 | Pençe S | 2017 | 100 | 100 | Bladder | Turkey | | Caucasian | CCS | MMP-9 | rs3918242 | TvsC |
| 91 | Wieczorek,E | 2014 | 241 | 199 | Bladder | Poland | | Caucasian | CCS | MMP-7 | rs11568818 | GvsA |
| 92 | Srivastava P | 2013 | 200 | 200 | Bladder | India | | Asian | CCS | MMP-2 | rs243865 | TvsC |
|  | Srivastava P | 2013 | 200 | 200 | Bladder | India | | Asian | CCS | MMP-2 | rs1053605 | TvsC |
| 93 | Srivastava,P | 2010 | 200 | 200 | Bladder | India | | Asian | CCS | MMP-7 | rs11568818 | GvsA |
| 94 | Srivastava P | 2010 | 200 | 200 | Bladder | India | | Asian | CCS | MMP-9 | rs17556 | QvsR |
|  | Srivastava P | 2010 | 200 | 200 | Bladder | India | | Asian | CCS | MMP-9 | rs2250889 | CvsG |
|  | Srivastava P | 2010 | 200 | 200 | Bladder | India | | Asian | CCS | MMP-9 | rs17577 | AvsG |
| 95 | Kader AK | 2007 | 560 | 560 | Bladder | USA | | Caucasian | CCS | MMP-2 | rs243865 | TvsC |
|  | Kader AK | 2007 | 560 | 560 | Bladder | USA | | Caucasian | CCS | MMP-9 | rs3918242 | TvsC |
|  | Kader AK | 2007 | 560 | 560 | Bladder | USA | | Caucasian | CCS | MMP-9 | rs17576 | GvsA |
|  | Kader AK | 2007 | 560 | 560 | Bladder | USA | | Caucasian | CCS | MMP-9 | rs2250889 | CvsG |
|  | Kader AK | 2007 | 560 | 560 | Bladder | USA | | Caucasian | CCS | MMP-9 | rs17577 | AvsG |
| 96 | Yu,C | 2002 | 781 | 852 | Lung | China | | Asian | CCS | MMP-2 | rs243865 | TvsC |
| 97 | Zhou,Y | 2005 | 770 | 777 | Lung | China | | Asian | CCS | MMP-2 | rs243865 | TvsC |
|  | Zhou,Y | 2005 | 770 | 777 | Lung | China | | Asian | CCS | MMP-2 | rs1053605 | TvsC |
| 98 | Wang Y | 2005 | 243 | 350 | Lung | China | | Asian | CCS | MMP-9 | rs3918242 | TvsC |
| 99 | Hu Z | 2005 | 744 | 747 | Lung | China | | Asian | CCS | MMP-9 | rs17576 | GvsA |
|  | Hu Z | 2005 | 744 | 747 | Lung | China | | Asian | CCS | MMP-9 | rs2250889 | CvsG |
|  | Hu Z | 2005 | 744 | 747 | Lung | China | | Asian | CCS | MMP-9 | rs17577 | AvsG |
| 100 | Chen,GL | 2019 | 358 | 716 | Lung | China | | Asian | CCS | MMP-2 | rs243865 | TvsC |
|  | Chen,GL | 2019 | 358 | 716 | Lung | China | | Asian | CCS | MMP-2 | rs1053605 | TvsC |
| 101 | Li W | 2019 | 245 | 258 | Lung | China | | Asian | CCS | MMP-9 | rs3918242 | TvsC |
| 102 | Chen,GL | 2018 | 358 | 716 | Lung | China | | Asian | CCS | MMP-7 | rs11568818 | GvsA |
|  | Chen GL | 2018 | 358 | 716 | Lung | China | | Asian | CCS | MMP-7 | rs11568819 | TvsC |
| 103 | Sanli,M | 2013 | 132 | 80 | Lung | Turkey | | Caucasian | CCS | MMP-2 | rs1053605 | TvsC |
|  | Sanli,M | 2013 | 132 | 80 | Lung | Turkey | | Caucasian | CCS | MMP-7 | rs11568818 | GvsA |
| 104 | González-Arriaga | 2012 | 879 | 803 | Lung | Spain | | Caucasian | CCS | MMP-9 | rs3918242 | TvsC |
|  | González-Arriaga | 2012 | 879 | 803 | Lung | Spain | | Caucasian | CCS | MMP-2 | rs1053605 | TvsC |
| 105 | Ayşegül,B | 2011 | 200 | 100 | Lung | Turkey | | Caucasian | CCS | MMP-2 | rs243865 | TvsC |
| 106 | Chiranjeevi,P | 2017 | 300 | 300 | Breast | India | | Asian | CCS | MMP-9 | rs3918242 | TvsC |
| 107 | Chou,AK | 2017 | 1232 | 1232 | Breast | China | | Asian | CCS | MMP-7 | rs11568818 | GvsA |
|  | Chou AK | 2017 | 1232 | 1232 | Breast | China | | Asian | CCS | MMP-7 | rs11568819 | TvsC |
| 108 | Yari,K | 2015 | 100 | 151 | Breast | Iran | | Asian | CCS | MMP-7 | rs11568818 | GvsA |
| 109 | Rahimi | 2015 | 101 | 104 | Breast | Iran | | Asian | CCS | MMP-9 | rs3918242 | TvsC |
| 110 | Ben Néjima D | 2015 | 210 | 250 | Breast | Tunisia | | African | CCS | MMP-2 | rs243865 | TvsC |
| 111 | Yari K | 2014 | 98 | 135 | Breast | Iran | | Asian | CCS | MMP-2 | rs1053605 | TvsC |
| 112 | Chiranjeevi,P | 2014 | 200 | 191 | Breast | India | | Asian | CCS | MMP-9 | rs3918242 | TvsC |
| 113 | Saeed HM | 2013 | 90 | 92 | Breast | Saudi | | Asian | CCS | MMP-2 | rs243865 | TvsC |
| 114 | Zagouri F | 2013 | 113 | 124 | Breast | Greece | | Caucasian | CCS | MMP-2 | rs243865 | TvsC |
| 115 | Delgado-Enciso I | 2008 | 90 | 96 | Breast | Mexico | | Latin American | CCS | MMP-2 | rs243865 | TvsC |
| 116 | Lei H | 2007 | 959 | 952 | Breast | Sweden | | Caucasian | CCS | MMP-2 | rs243865 | TvsC |
| 117 | Zhou Y | 2004 | 462 | 509 | Breast | China | | Asian | CCS | MMP-2 | rs243865 | TvsC |
| 118 | Jerome,R | 2007 | 90 | 90 | Lung | France | | Caucasian | CCS | MMP-2 | rs1053605 | TvsC |
|  | Jerome,R | 2007 | 90 | 90 | Lung | France | | Caucasian | CCS | MMP-2 | rs243865 | TvsC |
|  | Jerome,R | 2007 | 90 | 90 | Lung | France | | Caucasian | CCS | MMP-9 | rs3918242 | TvsC |
| 119 | Edyta Wieczorek | 2013 | 241 | 199 | Bladder | Poland | | Caucasian | CCS | MMP-2 | rs243865 | TvsC |
|  | Edyta Wieczorek | 2013 | 241 | 199 | Bladder | Poland | | Caucasian | CCS | MMP-9 | rs3918242 | TvsC |
| 120 | Ghilardi | 2003 | 58 | 111 | Colorectal | Italy | | Caucasian | CCS | MMP-7 | rs11568818 | GvsA |
| 121 | Kubben | 2006 | 79 | 169 | Gastric | Nederland | | Caucasian | CCS | MMP-7 | rs11568818 | GvsA |
|  | Kubben | 2006 | 79 | 169 | Gastric | Nederland | | Caucasian | CCS | MMP-7 | rs11568819 | TvsC |
|  | Kubben | 2006 | 79 | 169 | Gastric | Nederland | | Caucasian | CCS | MMP-2 | rs243865 | TvsC |
|  | Kubben | 2006 | 79 | 169 | Gastric | Nederland | | Caucasian | CCS | MMP-9 | rs3918242 | TvsC |
| 122 | Fang,WL | 2013 | 246 | 252 | Gastric | China | | Asian | CCS | MMP-7 | rs11568818 | CvsT |
| 123 | Aysegul Bayramoglu | 2009 | 200 | 100 | Lung | Turkey | | Caucasian | CCS | MMP-9 | rs3918242 | TvsC |
| 124 | Hakan,A | 2010 | 135 | 58 | Gastric | Germany | | Caucasian | CCS | MMP-2 | rs243865 | TvsC |
|  | Hakan,A | 2010 | 135 | 58 | Gastric | Germany | | Caucasian | CCS | MMP-7 | rs11568818 | GvsA |
| 125 | Holliday,DL | 2007 | 13 | 19 | Breast | UK | | Caucasian | CCS | MMP-9 | rs3918242 | TvsC |
| 126 | Roehe,AV | 2007 | 96 | 100 | Breast | Brazil | | Mixed | CCS | MMP-9 | rs3918242 | TvsC |
|  | Roehe AV | 2007 | 89 | 100 | Breast | Brazil | | Mixed | CCS | MMP-2 | rs243865 | TvsC |
| 127 | Chahil JK | 2014 | 80 | 80 | Breast | Malaysian | | Asian | CCS | MMP-9 | rs17576 | CvsA |
|  | Chahil JK | 2014 | 80 | 80 | Breast | Malaysian | | Asian | CCS | MMP-9 | rs2250889 | AvsG |
| 128 | [Eric J Jacobs](https://pubmed.ncbi.nlm.nih.gov/?term=Jacobs+EJ&cauthor_id=18398039) | 2008 | 1425 | 1453 | Prostate | USA | | Caucasian | CCS | MMP-2 | rs1477017 | GvsA |
|  | [Eric J Jacobs](https://pubmed.ncbi.nlm.nih.gov/?term=Jacobs+EJ&cauthor_id=18398039) | 2008 | 1425 | 1453 | Prostate | USA | | Caucasian | CCS | MMP-2 | rs17301608 | TvsC |
|  | [Eric J Jacobs](https://pubmed.ncbi.nlm.nih.gov/?term=Jacobs+EJ&cauthor_id=18398039) | 2008 | 1425 | 1453 | Prostate | USA | | Caucasian | CCS | MMP-2 | rs11639960 | AvsG |
| 129 | [Shunji Matsumura](https://pubmed.ncbi.nlm.nih.gov/?term=Matsumura+S&cauthor_id=15565457) | 2005 | 177 | 224 | Gastric | Japan | | Asian | CCS | MMP-9 | rs3918242 | TvsC |
| 130 | [Kinya Okamoto](https://pubmed.ncbi.nlm.nih.gov/?term=Okamoto+K&cauthor_id=20467172) | 2010 | 92 | 83 | Hepatocellular | Japan | | Asian | CCS | MMP-9 | rs3918242 | TvsC |
| 131 | Zhai | 2007 | 432 | 480 | Hepatocellular | China | | Asian | CCS | MMP-9 | rs3918242 | TvsC |
| 132 | Sugreev,V | 2015 | 230 | 233 | Gastric | India | | Asian | CCS | MMP-9 | rs3918242 | TvsC |
| 133 | Beeghly-Fadiel,A | 2008 | 1079 | 1082 | Breast | China | | Asian | CCS | MMP-7 | rs11568818 | GvsA |
|  | Beeghly-Fadiel A | 2008 | 1079 | 1082 | Breast | China | | Asian | CCS | MMP-7 | rs880197 | TvsA |
|  | Beeghly-Fadiel A | 2008 | 1079 | 1082 | Breast | China | | Asian | CCS | MMP-7 | rs17098318 | AvsG |
|  | Beeghly-Fadiel A | 2008 | 1079 | 1082 | Breast | China | | Asian | CCS | MMP-7 | rs11568819 | AvsG |
|  | Beeghly-Fadiel A | 2008 | 1079 | 1082 | Breast | China | | Asian | CCS | MMP-7 | rs11225307 | GvsA |
|  | Beeghly-Fadiel A | 2008 | 1079 | 1082 | Breast | China | | Asian | CCS | MMP-7 | rs17352054 | CvsA |
|  | Beeghly-Fadiel A | 2008 | 1079 | 1082 | Breast | China | | Asian | CCS | MMP-7 | rs10895304 | GvsA |
|  | Beeghly-Fadiel A | 2008 | 1079 | 1082 | Breast | China | | Asian | CCS | MMP-7 | rs7935378 | CvsT |
|  | Beeghly-Fadiel A | 2008 | 1079 | 1082 | Breast | China | | Asian | CCS | MMP-7 | rs12184413 | TvsC |
|  | Beeghly-Fadiel A | 2008 | 1079 | 1082 | Breast | China | | Asian | CCS | MMP-7 | rs11225297 | GvsA |
| 134 | Lei H | 2007 | 949 | 948 | Breast | German | | Caucasian | CCS | MMP-2 | rs243865 | TvsC |
|  | Lei,H | 2007 | 946 | 946 | Breast | German | | Caucasian | CCS | MMP-9 | rs3918242 | TvsC |
| 135 | Ohtani | 2009 | 119 | 67 | Colorectal | Japan | | Asian | CCS | MMP-7 | rs11568818 | GvsA |
|  | Ohtani | 2009 | 119 | 67 | Colorectal | Japan | | Asian | CCS | MMP-2 | rs243865 | TvsC |
|  | Ohtani | 2009 | 119 | 67 | Colorectal | Japan | | Asian | CCS | MMP-9 | rs3918242 | TvsC |

Abbreviation: CCS, case-control study.

Reference:

1. Miao, X., et al., A functional polymorphism in the matrix metalloproteinase-2 gene promoter (-1306C/T) is associated with risk of development but not metastasis of gastric cardia adenocarcinoma. Cancer Res, 2003. 63(14): p. 3987-90.

2. Wu, C.Y., et al., Clinicopathological significance of MMP-2 and TIMP-2 genotypes in gastric cancer. Eur J Cancer, 2007. 43(4): p. 799-808.

3. Sugimoto, M., et al., Polymorphisms of matrix metalloproteinase-7 and chymase are associated with susceptibility to and progression of gastric cancer in Japan. J Gastroenterol, 2008. 43(10): p. 751-61.

4. Liu, L., et al., Association of candidate genetic variations with gastric cardia adenocarcinoma in Chinese population: a multiple interaction analysis. Carcinogenesis, 2011. 32(3): p. 336-42.

5. Malik, M.A., S.A. Zargar, and B. Mittal, Role of the metalloproteinase-7 (181A>G) polymorphism in gastric cancer susceptibility: a case control study in Kashmir valley. Asian Pac J Cancer Prev, 2011. 12(1): p. 73-6.

6. Kim, J., et al., Lymph node metastasis of gastric cancer is associated with the interaction between poly (ADP-ribose) polymerase 1 and matrix metallopeptidase 2. DNA Cell Biol, 2011. 30(12): p. 1011-7.

7. Kesh, K., et al., Association of MMP7 -181A→G Promoter Polymorphism with Gastric Cancer Risk: INFLUENCE OF NICOTINE IN DIFFERENTIAL ALLELE-SPECIFIC TRANSCRIPTION VIA INCREASED PHOSPHORYLATION OF cAMP-RESPONSE ELEMENT-BINDING PROTEIN (CREB). J Biol Chem, 2015. 290(23): p. 14391-406.

8. Avcı, N., et al., Association and Prognostic Significance of the Functional -1562C/T Polymorphism in the Promoter Region of MMP-9 in Turkish Patients with Gastric Cancer. Pathol Oncol Res, 2015. 21(4): p. 1243-7.

9. Krishnaveni, D., et al., MMP 9 Gene Promoter Polymorphism in Gastric Cancer. Indian J Clin Biochem, 2012. 27(3): p. 259-64.

10. Zhang, D.Y., et al., Correlations of MMP-2 and TIMP-2 gene polymorphisms with the risk and prognosis of gastric cancer. Int J Clin Exp Med, 2015. 8(11): p. 20391-401.

11. Fu, C.K., et al., The Association of MMP7 Promoter Polymorphisms With Gastric Cancer. Anticancer Res, 2020. 40(2): p. 695-702.

12. Li, Y., et al., Association of functional polymorphisms in MMPs genes with gastric cardia adenocarcinoma and esophageal squamous cell carcinoma in high incidence region of North China. Mol Biol Rep, 2010. 37(1): p. 197-205.

13. Yu, C., et al., Functional haplotypes in the promoter of matrix metalloproteinase-2 predict risk of the occurrence and metastasis of esophageal cancer. Cancer Res, 2004. 64(20): p. 7622-8.

14. Wu, J., et al., Association of matrix metalloproteinases-9 gene polymorphisms with genetic susceptibility to esophageal squamous cell carcinoma. DNA Cell Biol, 2008. 27(10): p. 553-7.

15. Malik, M.A., et al., Association of matrix metalloproteinase-7 (-181A>G) polymorphism with risk of esophageal squamous cell carcinoma in Kashmir Valley. Saudi J Gastroenterol, 2011. 17(5): p. 301-6.

16. Guan, X., et al., Matrix metalloproteinase 1, 3, and 9 polymorphisms and esophageal squamous cell carcinoma risk. Med Sci Monit, 2014. 20: p. 2269-74.

17. Zhang, L., R.X. Xi, and X.Z. Zhang, Matrix metalloproteinase variants associated with risk and clinical outcome of esophageal cancer. Genet Mol Res, 2015. 14(2): p. 4616-24.

18. Eftekhary, H., et al., The influence of matrix metalloproteinase-2, -9, and -12 promoter polymorphisms on Iranian patients with oesophageal squamous cell carcinoma. Contemp Oncol (Pozn), 2015. 19(4): p. 300-5.

19. Wang, N., et al., MMP-2, -3 and TIMP-2, -3 polymorphisms in colorectal cancer in a Chinese Han population: A case-control study. Gene, 2020. 730: p. 144320.

20. Wu, M.H., et al., Association of Matrix Metalloproteinase-9 rs3918242 Promoter Genotypes With Colorectal Cancer Risk. Anticancer Res, 2019. 39(12): p. 6523-6529.

21. Yueh, T.C., et al., The Contribution of MMP-7 Genotypes to Colorectal Cancer Susceptibility in Taiwan. Cancer Genomics Proteomics, 2018. 15(3): p. 207-212.

22. Ben Néjima, D., et al., Clinicopathologic and Prognostic Significance of Gelatinase A in Tunisian Colorectal Cancer: A Case-Control Study. Appl Immunohistochem Mol Morphol, 2017. 25(1): p. 64-70.

23. Banday, M.Z., et al., Matrix metalloproteinase (MMP) -2, -7 and -9 promoter polymorphisms in colorectal cancer in ethnic Kashmiri population - A case-control study and a mini review. Gene, 2016. 589(1): p. 81-89.

24. Moreno-Ortiz, J.M., et al., Association of MMP7-181A/G and MMP13-77A/G polymorphisms with colorectal cancer in a Mexican population. Genet Mol Res, 2014. 13(2): p. 3537-44.

25. Saeed, H.M., et al., Matrix metalloproteinase-2 (-1306 c>t) promoter polymorphism and risk of colorectal cancer in the Saudi population. Asian Pac J Cancer Prev, 2013. 14(10): p. 6025-30.

26. Yang, Z.H., et al., MMP-9 polymorphisms are related to serum lipids levels but not associated with colorectal cancer susceptibility in Chinese population. Mol Biol Rep, 2012. 39(10): p. 9399-404.

27. Dziki, L., et al., A/G Polymorphism of the MMP-7 Gene Promoter Region in Colorectal Cancer. Pol Przegl Chir, 2011. 83(11): p. 622-6.

28. Kang, M.J., et al., Associations between single nucleotide polymorphisms of MMP2, VEGF, and HIF1A genes and the risk of developing colorectal cancer. Anticancer Res, 2011. 31(2): p. 575-84.

29. Park, K.S., et al., Clinical characteristics of TIMP2, MMP2, and MMP9 gene polymorphisms in colorectal cancer. J Gastroenterol Hepatol, 2011. 26(2): p. 391-7.

30. Fang, W.L., et al., Association of matrix metalloproteinases 1, 7, and 9 gene polymorphisms with genetic susceptibility to colorectal carcinoma in a Han Chinese population. DNA Cell Biol, 2010. 29(11): p. 657-61.

31. de Lima, J.M., et al., E-cadherin and metalloproteinase-1 and -7 polymorphisms in colorectal cancer. Int J Biol Markers, 2009. 24(2): p. 99-106.

32. Xing, L.L., et al., Matrix metalloproteinase-9-1562C>T polymorphism may increase the risk of lymphatic metastasis of colorectal cancer. World J Gastroenterol, 2007. 13(34): p. 4626-9.

33. Woo, M., et al., Clinical implications of matrix metalloproteinase-1, -3, -7, -9, -12, and plasminogen activator inhibitor-1 gene polymorphisms in colorectal cancer. J Gastroenterol Hepatol, 2007. 22(7): p. 1064-70.

34. Lièvre, A., et al., Genetic polymorphisms of MMP1, MMP3 and MMP7 gene promoter and risk of colorectal adenoma. BMC Cancer, 2006. 6: p. 270.

35. Elander, N., P. Söderkvist, and K. Fransén, Matrix metalloproteinase (MMP) -1, -2, -3 and -9 promoter polymorphisms in colorectal cancer. Anticancer Res, 2006. 26(1b): p. 791-5.

36. Xu, E., et al., A single nucleotide polymorphism in the matrix metalloproteinase-2 promoter is associated with colorectal cancer. Biochem Biophys Res Commun, 2004. 324(3): p. 999-1003.

37. El Samanoudy, A., et al., Matrix metalloproteinase-9 gene polymorphism in hepatocellular carcinoma patients with hepatitis B and C viruses. Genet Mol Res, 2014. 13(3): p. 8025-34.

38. Qiu, W., et al., No association of MMP-7, MMP-8, and MMP-21 polymorphisms with the risk of hepatocellular carcinoma in a Chinese population. Cancer Epidemiol Biomarkers Prev, 2008. 17(9): p. 2514-8.

39. Sharma, K.L., et al., Higher risk of matrix metalloproteinase (MMP-2, 7, 9) and tissue inhibitor of metalloproteinase (TIMP-2) genetic variants to gallbladder cancer. Liver Int, 2012. 32(8): p. 1278-86.

40. Hsu, S.W., et al., Contribution of Matrix Metalloproteinase-2 Promoter Genotypes to Nasopharyngeal Cancer Susceptibility and Metastasis in Taiwan. Cancer Genomics Proteomics, 2019. 16(4): p. 287-292.

41. Shao, J.Y., et al., A single nucleotide polymorphism in the matrix metalloproteinase 2 promoter is closely associated with high risk of nasopharyngeal carcinoma in Cantonese from southern China. Chin J Cancer, 2011. 30(9): p. 620-6.

42. Zhou, G., et al., Functional polymorphisms and haplotypes in the promoter of the MMP2 gene are associated with risk of nasopharyngeal carcinoma. Hum Mutat, 2007. 28(11): p. 1091-7.

43. Nasr, H.B., et al., Matrix metalloproteinase-1 (-1607) 1G/2G and -9 (-1562) C/T promoter polymorphisms: susceptibility and prognostic implications in nasopharyngeal carcinomas. Clin Chim Acta, 2007. 384(1-2): p. 57-63.

44. Vairaktaris, E., et al., Gene polymorphisms related to angiogenesis, inflammation and thrombosis that influence risk for oral cancer. Oral Oncol, 2009. 45(3): p. 247-53.

45. Tsai, C.W., et al., Contribution of MMP2 Promoter Genotypes to Oral Cancer Susceptibility, Recurrence and Metastasis in Taiwan. Anticancer Res, 2018. 38(12): p. 6821-6826.

46. Shih, L.C., et al., Association of Matrix Metalloproteinase-7 Genotypes to the Risk of Oral Cancer in Taiwan. Anticancer Res, 2018. 38(4): p. 2087-2092.

47. Yete, S., S. Pradhan, and D. Saranath, Single nucleotide polymorphisms in an Indian cohort and association of CNTN4, MMP2 and SNTB1 variants with oral cancer. Cancer Genet, 2017. 214-215: p. 16-25.

48. Chaudhary, A.K., et al., Role of functional polymorphism of matrix metalloproteinase-2 (-1306 C/T and -168 G/T) and MMP-9 (-1562 C/T) promoter in oral submucous fibrosis and head and neck squamous cell carcinoma in an Indian population. Biomarkers, 2011. 16(7): p. 577-86.

49. Vairaktaris, E., et al., High gene expression of matrix metalloproteinase-7 is associated with early stages of oral cancer. Anticancer Res, 2007. 27(4b): p. 2493-8.

50. Tu, H.F., et al., Functional -1562 C-to-T polymorphism in matrix metalloproteinase-9 (MMP-9) promoter is associated with the risk for oral squamous cell carcinoma in younger male areca users. J Oral Pathol Med, 2007. 36(7): p. 409-14.

51. Lin, S.C., et al., Functional genotype in matrix metalloproteinases-2 promoter is a risk factor for oral carcinogenesis. J Oral Pathol Med, 2004. 33(7): p. 405-9.

52. Zhang, H., et al., MMP-2 and MMP-9 gene polymorphisms associated with cervical cancer risk. Int J Clin Exp Pathol, 2017. 10(12): p. 11760-11765.

53. Singh, N., et al., The protective role of the -1306C>T functional polymorphism in matrix metalloproteinase-2 gene is associated with cervical cancer: implication of human papillomavirus infection. Tumour Biol, 2016. 37(4): p. 5295-303.

54. Xie, B., et al., Genetic polymorphisms in MMP 2, 3, 7, and 9 genes and the susceptibility and clinical outcome of cervical cancer in a Chinese Han population. Tumour Biol, 2016. 37(4): p. 4883-8.

55. Srivastava, P., et al., No Association of Matrix Metalloproteinase [MMP]-2 (-735C > T) and Tissue Inhibitor of Metalloproteinase [TIMP]-2 (-418G > C) Gene Polymorphisms with Cervical Cancer Susceptibility. Indian J Clin Biochem, 2013. 28(1): p. 13-8.

56. Wu, S., et al., Correlation of polymorphism of IL-8 and MMP-7 with occurrence and lymph node metastasis of early stage cervical cancer. J Huazhong Univ Sci Technolog Med Sci, 2011. 31(1): p. 114-119.

57. Singh, H., M. Jain, and B. Mittal, MMP-7 (-181A>G) promoter polymorphisms and risk for cervical cancer. Gynecol Oncol, 2008. 110(1): p. 71-5.

58. Baltazar-Rodriguez, L.M., et al., Polymorphism in the matrix metalloproteinase-2 gene promoter is associated with cervical neoplasm risk in Mexican women. Biochem Genet, 2008. 46(3-4): p. 137-44.

59. Yi, Y.C., et al., Matrix metalloproteinase-7 (MMP-7) polymorphism is a risk factor for endometrial cancer susceptibility. Clin Chem Lab Med, 2010. 48(3): p. 337-44.

60. Wang, Y., et al., Genetic variants in matrix metalloproteinase genes as disposition factors for ovarian cancer risk, survival, and clinical outcome. Mol Carcinog, 2015. 54(6): p. 430-9.

61. Li, Y., et al., Polymorphisms in the promoter regions of the matrix metalloproteinases-1, -3, -7, and -9 and the risk of epithelial ovarian cancer in China. Gynecol Oncol, 2006. 101(1): p. 92-6.

62. Roncevic, J., et al., MMP-9-1562 C/T single nucleotide polymorphism associates with increased MMP-9 level and activity during papillary thyroid carcinoma progression. Pathology, 2019. 51(1): p. 55-61.

63. Nan, H., et al., Missense polymorphisms in matrix metalloproteinase genes and skin cancer risk. Cancer Epidemiol Biomarkers Prev, 2008. 17(12): p. 3551-7.

64. Radunovic, M., et al., The MMP-2 and MMP-9 promoter polymorphisms and susceptibility to salivary gland cancer. J buon, 2016. 21(3): p. 597-602.

65. Strbac, D., et al., Matrix Metalloproteinases Polymorphisms as Baseline Risk Predictors in Malignant Pleural Mesothelioma. Radiol Oncol, 2018. 52(2): p. 160-166.

66. Lin, C.M., et al., The Relationship Between MMP-2 -1306C>T and MMP-9 -1562C>T Polymorphisms and the Risk and Prognosis of T-Cell Acute Lymphoblastic Leukemia in a Chinese Population: A Case-Control Study. Cell Physiol Biochem, 2017. 42(4): p. 1458-1468.

67. Gouda, H.M., et al., Association between matrix metalloproteinase 2 (MMP2) promoter polymorphisms and the susceptibility to non-Hodgkin's lymphoma in Egyptians. Ann Hematol, 2014. 93(8): p. 1313-8.

68. Vasku, A., et al., Matrix metalloproteinase-2 promoter genotype as a marker of cutaneous T-cell lymphoma early stage. J Biomed Biotechnol, 2010. 2010: p. 805907.

69. Liutkevicius, V., et al., Matrix Metalloproteinases (MMP-2,-3,-9) Gene Polymorphisms in Cases of Benign Vocal Fold Lesions and Laryngeal Carcinoma. In Vivo, 2020. 34(1): p. 267-274.

70. P, O.C. and P. Khantapura, The role of genetic polymorphisms in the promoters of the matrix metalloproteinase-2 and tissue inhibitor of metalloproteinase-2 genes in head and neck cancer. Oral Oncol, 2006. 42(3): p. 257-67.

71. Ozden, M., et al., Polymorphisms in the Matrix Metalloproteinase-9 Promoters and Susceptibility to Glial Tumors in Turkey. Turk Neurosurg, 2017. 27(5): p. 690-695.

72. Kawal, P., et al., Correlations of polymorphisms in matrix metalloproteinase-1, -2, and -7 promoters to susceptibility to malignant gliomas. Asian J Neurosurg, 2016. 11(2): p. 160-6.

73. Kumar, R., et al., Matrix metalloproteinase-2 gene polymorphism is not associated with increased glioblastoma multiforme susceptibility: an Indian institutional experience. Neurol India, 2011. 59(2): p. 236-40.

74. Lu, Z., et al., Polymorphisms in the matrix metalloproteinase-1, 3, and 9 promoters and susceptibility to adult astrocytoma in northern China. J Neurooncol, 2007. 85(1): p. 65-73.

75. Lu, Z., et al., Association between the functional polymorphism in the matrix metalloproteinase-7 promoter and susceptibility to adult astrocytoma. Brain Res, 2006. 1118(1): p. 6-12.

76. Pei, J.S., et al., Contribution of Matrix Metalloproteinase-7 Genotypes to the Risk of Non-solid Tumor, Childhood Leukemia. Anticancer Res, 2017. 37(12): p. 6679-6684.

77. Pirooz, H.J., et al., Functional SNP in microRNA-491-5p binding site of MMP9 3'-UTR affects cancer susceptibility. J Cell Biochem, 2018. 119(7): p. 5126-5134.

78. Zhang, J., et al., The functional polymorphism in the matrix metalloproteinase-7 promoter increases susceptibility to esophageal squamous cell carcinoma, gastric cardiac adenocarcinoma and non-small cell lung carcinoma. Carcinogenesis, 2005. 26(10): p. 1748-53.

79. Liao, C.H., et al., The Contribution of MMP-7 Promoter Polymorphisms in Renal Cell Carcinoma. In Vivo, 2017. 31(4): p. 631-635.

80. Awakura, Y., et al., Matrix metalloproteinase-9 polymorphisms and renal cell carcinoma in a Japanese population. Cancer Lett, 2006. 241(1): p. 59-63.

81. Białkowska, K., et al., Association of zinc level and polymorphism in MMP-7 gene with prostate cancer in Polish population. PLoS One, 2018. 13(7): p. e0201065.

82. Shajarehpoor Salavati, L., F. Tafvizi, and H.K. Manjili, The association between MMP2 -1306 C > T (rs243865) polymorphism and risk of prostate cancer. Ir J Med Sci, 2017. 186(1): p. 103-111.

83. Adabi, Z., et al., Genetic Polymorphism of MMP2 Gene and Susceptibility to Prostate Cancer. Arch Med Res, 2015. 46(7): p. 546-50.

84. Yaykaşli, K.O., et al., Polymorphisms in MMP-2 and TIMP-2 in Turkish patients with prostate cancer. Turk J Med Sci, 2014. 44(5): p. 839-43.

85. Srivastava, P., et al., Association of promoter polymorphisms in MMP2 and TIMP2 with prostate cancer susceptibility in North India. Arch Med Res, 2012. 43(2): p. 117-24.

86. Dos Reis, S.T., et al., Genetic polymorphisms of matrix metalloproteinases: susceptibility and prognostic implications for prostate cancer. J Urol, 2009. 181(5): p. 2320-5.

87. Sfar, S., et al., TSP1 and MMP9 genetic variants in sporadic prostate cancer. Cancer Genet Cytogenet, 2007. 172(1): p. 38-44.

88. Mao, F., et al., The association between matrix metalloproteinase-7 genetic variant and bladder cancer risk in a Chinese Han population. Clin Exp Med, 2019. 19(4): p. 565-570.

89. Liao, C.H., et al., Association of Matrix Metalloproteinase-7 Genotypes with the Risk of Bladder Cancer. In Vivo, 2018. 32(5): p. 1045-1050.

90. Pençe, S., et al., rs3918242 variant genotype frequency and increased TIMP-2 and MMP-9 expression are positively correlated with cancer invasion in urinary bladder cancer. Cell Mol Biol (Noisy-le-grand), 2017. 63(9): p. 46-52.

91. Wieczorek, E., et al., MMP7 and MMP8 genetic polymorphisms in bladder cancer patients. Cent European J Urol, 2014. 66(4): p. 405-10.

92. Srivastava, P., R. Kapoor, and R.D. Mittal, Association of single nucleotide polymorphisms in promoter of matrix metalloproteinase-2, 8 genes with bladder cancer risk in Northern India. Urol Oncol, 2013. 31(2): p. 247-54.

93. Srivastava, P., et al., Bladder cancer risk associated with genotypic polymorphism of the matrix metalloproteinase-1 and 7 in North Indian population. Dis Markers, 2010. 29(1): p. 37-46.

94. Srivastava, P., et al., Role of MMP-3 and MMP-9 and their haplotypes in risk of bladder cancer in North Indian cohort. Ann Surg Oncol, 2010. 17(11): p. 3068-75.

95. Kader, A.K., et al., Matrix metalloproteinase polymorphisms and bladder cancer risk. Cancer Res, 2006. 66(24): p. 11644-8.

96. Yu, C., et al., Correlation between a single nucleotide polymorphism in the matrix metalloproteinase-2 promoter and risk of lung cancer. Cancer Res, 2002. 62(22): p. 6430-3.

97. Zhou, Y., et al., Functional haplotypes in the promoter of matrix metalloproteinase-2 and lung cancer susceptibility. Carcinogenesis, 2005. 26(6): p. 1117-21.

98. Wang, Y., et al., No association between the C-1562T polymorphism in the promoter of matrix metalloproteinase-9 gene and non-small cell lung carcinoma. Lung Cancer, 2005. 49(2): p. 155-61.

99. Hu, Z., et al., Functional polymorphisms of matrix metalloproteinase-9 are associated with risk of occurrence and metastasis of lung cancer. Clin Cancer Res, 2005. 11(15): p. 5433-9.

100. Chen, G.L., et al., The association of matrix metalloproteinas-2 promoter polymorphisms with lung cancer susceptibility in Taiwan. Chin J Physiol, 2019. 62(5): p. 210-216.

101. Li, W., et al., Association of MMP9-1562C/T and MMP13-77A/G Polymorphisms with Non-Small Cell Lung Cancer in Southern Chinese Population. Biomolecules, 2019. 9(3).

102. Chen, G.L., et al., The Contribution of MMP-7 Promoter Polymorphisms to Taiwan Lung Cancer Susceptibility. Anticancer Res, 2018. 38(10): p. 5671-5677.

103. Sanli, M., et al., The relationship of metalloproteinase gene polymorphisms and lung cancer. J Surg Res, 2013. 183(2): p. 517-23.

104. González-Arriaga, P., et al., Genetic polymorphisms in MMP 2, 9 and 3 genes modify lung cancer risk and survival. BMC Cancer, 2012. 12: p. 121.

105. Ayşegül, B., et al., Is a single nucleotide polymorphism a risk factor for lung cancer in the matrix metalloproteinase-2 promoter? Mol Biol Rep, 2011. 38(3): p. 1469-74.

106. Padala, C., et al., Synergistic effect of collagenase-1 (MMP1), stromelysin-1 (MMP3) and gelatinase-B (MMP9) gene polymorphisms in breast cancer. PLoS One, 2017. 12(9): p. e0184448.

107. Chou, A.K., et al., The Contribution of Matrix Metalloproteinase-7 Promoter Genotypes in Breast Cancer in Taiwan. Anticancer Res, 2017. 37(9): p. 4973-4977.

108. Yari, K., et al., MMP-7 A-181G Polymorphism in Breast Cancer Patients from Western Iran. Breast Care (Basel), 2015. 10(6): p. 398-402.

109. Rahimi, Z., K. Yari, and Z. Rahimi, Matrix metalloproteinase-9 -1562T allele and its combination with MMP-2 -735 C allele are risk factors for breast cancer. Asian Pac J Cancer Prev, 2015. 16(3): p. 1175-9.

110. Ben Néjima, D., et al., Prognostic impact of polymorphism of matrix metalloproteinase-2 and metalloproteinase tissue inhibitor-2 promoters in breast cancer in Tunisia: case-control study. Tumour Biol, 2015. 36(5): p. 3815-22.

111. Yari, K., et al., The MMP-2 -735 C allele is a risk factor for susceptibility to breast cancer. Asian Pac J Cancer Prev, 2014. 15(15): p. 6199-203.

112. Chiranjeevi, P., et al., Gelatinase B (-1562C/T) polymorphism in tumor progression and invasion of breast cancer. Tumour Biol, 2014. 35(2): p. 1351-6.

113. Saeed, H.M., et al., Matrix metalloproteinase-2 C(-1306)T promoter polymorphism and breast cancer risk in the Saudi population. Acta Biochim Pol, 2013. 60(3): p. 405-9.

114. Zagouri, F., et al., MMP-2 -1306C>T polymorphism in breast cancer: a case-control study in a South European population. Mol Biol Rep, 2013. 40(8): p. 5035-40.

115. Delgado-Enciso, I., et al., Matrix metalloproteinase-2 promoter polymorphism is associated with breast cancer in a Mexican population. Gynecol Obstet Invest, 2008. 65(1): p. 68-72.

116. Gerger, A., et al., A multigenic approach to predict breast cancer risk. Breast Cancer Res Treat, 2007. 104(2): p. 159-64.

117. Zhou, Y., et al., Substantial reduction in risk of breast cancer associated with genetic polymorphisms in the promoters of the matrix metalloproteinase-2 and tissue inhibitor of metalloproteinase-2 genes. Carcinogenesis, 2004. 25(3): p. 399-404.

118. Rollin, J., et al., Influence of MMP-2 and MMP-9 promoter polymorphisms on gene expression and clinical outcome of non-small cell lung cancer. Lung Cancer, 2007. 56(2): p. 273-80.

119. Wieczorek, E., et al., Genetic polymorphisms in matrix metalloproteinases (MMPs) and tissue inhibitors of MPs (TIMPs), and bladder cancer susceptibility. BJU Int, 2013. 112(8): p. 1207-14.

120. Ghilardi, G., et al., Colorectal carcinoma susceptibility and metastases are associated with matrix metalloproteinase-7 promoter polymorphisms. Clin Chem, 2003. 49(11): p. 1940-2.

121. Kubben, F.J., et al., Clinical impact of MMP and TIMP gene polymorphisms in gastric cancer. Br J Cancer, 2006. 95(6): p. 744-51.

122. Fang, W.L., et al., Genetic polymorphisms in Matrix Metalloproteinases -1 and -7 and susceptibility to gastric cancer: an association study and meta-analysis. Iran J Allergy Asthma Immunol, 2013. 12(3): p. 203-10.

123. Bayramoglu, A., et al., The association of MMP-9 enzyme activity, MMP-9 C1562T polymorphism, and MMP-2 and -9 and TIMP-1, -2, -3, and -4 gene expression in lung cancer. Genet Test Mol Biomarkers, 2009. 13(5): p. 671-8.

124. Alakus, H., et al., Clinical impact of MMP and TIMP gene polymorphisms in gastric cancer. World J Surg, 2010. 34(12): p. 2853-9.

125. Holliday, D.L., et al., Intrinsic genetic characteristics determine tumor-modifying capacity of fibroblasts: matrix metalloproteinase-3 5A/5A genotype enhances breast cancer cell invasion. Breast Cancer Res, 2007. 9(5): p. R67.

126. Roehe, A.V., et al., Detection of polymorphisms in the promoters of matrix metalloproteinases 2 and 9 genes in breast cancer in South Brazil: preliminary results. Breast Cancer Res Treat, 2007. 102(1): p. 123-4.

127. Chahil, J.K., et al., Genetic polymorphisms associated with breast cancer in malaysian cohort. Indian J Clin Biochem, 2015. 30(2): p. 134-9.

128. Jacobs, E.J., et al., Polymorphisms in angiogenesis-related genes and prostate cancer. Cancer Epidemiol Biomarkers Prev, 2008. 17(4): p. 972-7.

129. Matsumura, S., et al., A single nucleotide polymorphism in the MMP-9 promoter affects tumor progression and invasive phenotype of gastric cancer. J Cancer Res Clin Oncol, 2005. 131(1): p. 19-25.

130. Okamoto, K., et al., The genotypes of IL-1 beta and MMP-3 are associated with the prognosis of HCV-related hepatocellular carcinoma. Intern Med, 2010. 49(10): p. 887-95.

131. Zhai, Y., et al., Functional polymorphisms in the promoters of MMP-1, MMP-2, MMP-3, MMP-9, MMP-12 and MMP-13 are not associated with hepatocellular carcinoma risk. Gut, 2007. 56(3): p. 445-7.

132. Verma, S., et al., An Overview of Matrix Metalloproteinase 9 Polymorphism and Gastric Cancer Risk. Asian Pac J Cancer Prev, 2015. 16(17): p. 7393-400.

133. Beeghly-Fadiel, A., et al., Common MMP-7 polymorphisms and breast cancer susceptibility: a multistage study of association and functionality. Cancer Res, 2008. 68(15): p. 6453-9.

134. Lei, H., et al., Promoter polymorphisms in matrix metalloproteinases and their inhibitors: few associations with breast cancer susceptibility and progression. Breast Cancer Res Treat, 2007. 103(1): p. 61-9.

135. Ohtani, H., N. Maeda, and Y.J.Y.A.M. Murawaki, Functional Polymorphisms in the Promoter Regions of Matrix Metalloproteinase-2, -3, -7, -9 and TNF-alpha Genes, and the Risk of Colorectal Neoplasm in Japanese. 2009. 52(1): p. 47-56.
