# Supplementary material for: Epidemiological Evidence Between Variants in Matrix Metalloproteinases-2, -7, and -9 and Cancer Risk
Source: Front Oncol. 2022 Apr 28;12:856831. doi: 10.3389/fonc.2022.856831 (PMC9095957; doi:10.3389/fonc.2022.856831)
Supplement: Supplementary file 4 [file Table_3.docx]

| Supplementary Table S3: Variants in MMPs showing no relation to cancer risk in meta-analyses with at least 2000 cases and 2000 controls in additive model | | | | | | | | | | | | | | | | |
| --- | --- | --- | --- | --- | --- | --- | --- | --- | --- | --- | --- | --- | --- | --- | --- | --- |
| **Gene** | **rs number** | **Alleles** | **cancer site** | **Ethnicity** | **MAF** | **Number Evaluation** | | **Risk of Meta-Analysis** | | | | | |  |  |  |
|  |  |  |  |  |  | **Studies** | **Sample size (case/controls)** | **Genetic Models** | **Effect model** | **OR (95%CI)** | ***P*_value_** | ***I^2^*** | ***P*_Q_** | **Power (%)** | **0.2** | **0.1** |
| MMP2 | rs243865 | TvsC | Breast | Overall | 0.2463 | 8 | 6019 (2952/3067) | Allelic | Random | 0.882 (0.678 -1.148) | 0.351 | 85.6 | 0.000 | 91.4 | 87.3 | 64.8 |
| MMP2 | rs243865 | TvsC | Breast | Overall | 0.2463 | 8 | 6019 (2952/3067) | Dominant | Random | 0.836 (0.612-1.140) | 0.257 | 84.9 | 0.000 | 76.1 | 73.9 | 57.6 |
| MMP2 | rs243865 | TvsC | Breast | Overall | 0.2463 | 8 | 6019 (2952/3067) | Recessive | Random | 0.940 (0.648-1.364) | 0.097 | 44.600 | 0.081 | 26.2 | 19.2 | 8.6 |
| MMP2 | rs243865 | TvsC | Breast | Caucasian | 0.2571 | 3 | 4031 (2011/2020) | Allelic | Fixed | 1.028 (0.931-1.136) | 0.580 | 50.500 | 0.132 | 79.5 | 72.5 | 48.9 |
| MMP2 | rs243865 | TvsC | Breast | Caucasian | 0.2571 | 3 | 4031 (2011/2020) | Dominant | Fixed | 1.029 (0.909-1.165) | 0.650 | 31.200 | 0.234 | 59.8 | 57.4 | 42.7 |
| MMP2 | rs243865 | TvsC | Breast | Caucasian | 0.2571 | 3 | 4031 (2011/2020) | Recessive | Fixed | 1.057 (0.832-1.341) | 0.650 | 10.700 | 0.327 | 20.6 | 14.6 | 7.4 |
| MMP2 | rs1053605 | TvsC | Lung | Overall | 0.1917 | 5 | 4426 (2165/2261) | Allelic | Random | 0.885 (0.697-1.123) | 0.314 | 66.3 | 0.018 | 74.3 | 75.6 | 51.8 |
| MMP2 | rs1053605 | TvsC | Lung | Overall | 0.1917 | 5 | 4426 (2165/2261) | Dominant | Random | 0.899 (0.633-1.276) | 0.551 | 78.7 | 0.001 | 59.9 | 60.5 | 45.3 |
| MMP2 | rs1053605 | TvsC | Lung | Overall | 0.1917 | 5 | 4426 (2165/2261) | Recessive | Fixed | 0.740 (0.531-1.030) | 0.074 | 0 | 0.65 | 14.5 | 15.4 | 7.6 |
| MMP7 | rs11568818 | CvsT | Breast | Asian | 0.0911 | 3 | 4876 (2411/2465) | Allelic | Random | 1.160 (0.894-1.505) | 0.263 | 67.4 | 0.047 | 52.8 | 80.0 | 56.2 |
| MMP7 | rs11568818 | CvsT | Breast | Asian | 0.0911 | 3 | 4876 (2411/2465) | Dominant | Fixed | 1.153 (0.985-1.349) | 0.077 | 20.9 | 0.282 | 46.8 | 65.2 | 49.4 |
| MMP7 | rs11568818 | CvsT | Breast | Asian | 0.0911 | 3 | 4876 (2411/2465) | Recessive | Random | 1.475 (0.616-3.530) | 0.383 | 68.7 | 0.041 | 7.4 | 16.6 | 7.9 |

Abbreviations: C, cytosine; T, thymine; OR, odds ratio; CI, confidence interval; MAF, minor allelic frequency in control.
