# Supplementary material for: Epidemiological Evidence Between Variants in Matrix Metalloproteinases-2, -7, and -9 and Cancer Risk
Source: Front Oncol. 2022 Apr 28;12:856831. doi: 10.3389/fonc.2022.856831 (PMC9095957; doi:10.3389/fonc.2022.856831)
Supplement: Supplementary file 5 [file Table_4.docx]

Supplementary Table S4： Associations between variants in the MMP-2, -7 and -9 and cancer risk.

| **Gene** | **Variant** | **Alleles** | **Cancer site** | **Ethnicity** | **MAF^a^** | **Number Evaluation** | | **Risk of Meta-Analysis** | | | | | | | **Venice Criteria^b^** | **FPRP^c^** | **Credibility of Evidence** |
| --- | --- | --- | --- | --- | --- | --- | --- | --- | --- | --- | --- | --- | --- | --- | --- | --- | --- |
|  |  |  |  |  |  | **N** | **Sample size (cases/controls)** | **Genetic Models** | **Effect model** | | **OR (95%CI)** | ***P*_value_** | ***I^2^*** | **P_Q_** |  |  |  |
| MMP2 | rs243865 | TvsC | Bladder | Overall | 0.2256 | 3 | 1928 (984/944) | Allelic | | Random | 1.242 (0.916-1.685) | 0.163 | 72.2 | 0.027 |  |  |  |
| MMP2 | rs243865 | TvsC | Bladder | Overall | 0.2256 | 3 | 1928 (984/944) | Dominant | | Random | 1.255 (0.920-1.712) | 0.152 | 60.8 | 0.078 |  |  |  |
| MMP2 | rs243865 | TvsC | Bladder | Overall | 0.2256 | 3 | 1928 (984/944) | Recessive | | Random | 1.487 (0.728-3.036) | 0.276 | 61.0 | 0.077 |  |  |  |
| MMP2 | rs243865 | TvsC | Breast | Overall | 0.2463 | 8 | 6019 (2952/3067) | Allelic | | Random | 0.882 (0.678-1.148) | 0.351 | 85.6 | 0.000 |  |  |  |
| MMP2 | rs243865 | TvsC | Breast | Overall | 0.2463 | 8 | 6019 (2952/3067) | Dominant | | Random | 0.836 (0.612-1.140) | 0.257 | 84.9 | 0.000 |  |  |  |
| MMP2 | rs243865 | TvsC | Breast | Overall | 0.2463 | 8 | 6019 (2952/3067) | Recessive | | Random | 0.940 (0.648-1.364) | 0.097 | 44.6 | 0.081 |  |  |  |
| MMP2 | rs243865 | TvsC | Breast | Caucasian | 0.2571 | 3 | 4031 (2011/2020) | Allelic | | Fixed | 1.028 (0.931-1.136) | 0.580 | 50.5 | 0.132 |  |  |  |
| MMP2 | rs243865 | TvsC | Breast | Caucasian | 0.2571 | 3 | 4031 (2011/2020) | Dominant | | Fixed | 1.029 (0.909-1.165) | 0.650 | 31.2 | 0.234 |  |  |  |
| MMP2 | rs243865 | TvsC | Breast | Caucasian | 0.2571 | 3 | 4031 (2011/2020) | Recessive | | Fixed | 1.057 (0.832-1.341) | 0.650 | 10.7 | 0.327 |  |  |  |
| MMP2 | rs243865 | TvsC | Colorectal | Overall | 0.2042 | 7 | 1676 (775/901) | Allelic | | Random | 0.865 (0.511-1.465) | 0.590 | 85.7 | 0.000 |  |  |  |
| MMP2 | rs243865 | TvsC | Colorectal | Overall | 0.2042 | 7 | 1676 (775/901) | Dominant | | Random | 0.763 (0.387-1.502) | 0.433 | 87.5 | 0.000 |  |  |  |
| MMP2 | rs243865 | TvsC | Colorectal | Overall | 0.2042 | 7 | 1676 (775/901) | Recessive | | Fixed | 0.880 (0.582-1.329) | 0.542 | 41.5 | 0.128 |  |  |  |
| MMP2 | rs243865 | TvsC | Colorectal | Asian | 0.1674 | 4 | 864 (437/427) | Allelic | | Random | 0.887 (0.496-1.586) | 0.685 | 67.8 | 0.025 |  |  |  |
| MMP2 | rs243865 | TvsC | Colorectal | Asian | 0.1674 | 4 | 864 (437/427) | Dominant | | Random | 0.837 (0.466-1.504) | 0.552 | 55.8 | 0.079 |  |  |  |
| MMP2 | rs243865 | TvsC | Colorectal | Asian | 0.1674 | 4 | 864 (437/427) | Recessive | | Fixed | 0.644 (0.364-1.138) | 0.130 | 20.8 | 0.283 |  |  |  |
| MMP2 | rs243865 | TvsC | Colorectal | Caucasian | 0.2658 | 3 | 818 (338/480) | Allelic | | Random | 0.794 (0.287-2.200) | 0.658 | 93.9 | 0.000 |  |  |  |
| MMP2 | rs243865 | TvsC | Colorectal | Caucasian | 0.2658 | 3 | 818 (338/480) | Dominant | | Random | 0.604 (0.151-2.427) | 0.478 | 95.1 | 0.000 |  |  |  |
| MMP2 | rs243865 | TvsC | Colorectal | Caucasian | 0.2658 | 3 | 818 (338/480) | Recessive | | Fixed | 1.256 (0.686-2.299) | 0.460 | 49.2 | 0.140 |  |  |  |
| MMP2 | rs243865 | TvsC | Esophageal | Asian | 0.1565 | 4 | 2850 (1157/1693) | Allelic | | Fixed | 0.751 (0.643-0.877) | < 0.001 | 41.4 | 0.163 | BBA | 0.006 | strong |
| MMP2 | rs243865 | TvsC | Esophageal | Asian | 0.1565 | 4 | 2850 (1157/1693) | Dominant | | Fixed | 0.723 (0.607-0.862) | < 0.001 | 46.9 | 0.13 | BBA | 0.007 | strong |
| MMP2 | rs243865 | TvsC | Esophageal | Asian | 0.1565 | 4 | 2850 (1157/1693) | Recessive | | Fixed | 0.714 (0.436-1.169) | 0.181 | 0.0 | 0.406 |  |  |  |
| MMP2 | rs243865 | TvsC | Gastric | Overall | 0.1479 | 8 | 4647 (1818/2829) | Allelic | | Random | 0.723 (0.501-1.042) | 0.082 | 85.9 | 0 |  |  |  |
| MMP2 | rs243865 | TvsC | Gastric | Overall | 0.1479 | 8 | 4647 (1818/2829) | Dominant | | Random | 0.680 (0.462-1.001) | 0.05 | 84.5 | 0 |  |  |  |
| MMP2 | rs243865 | TvsC | Gastric | Overall | 0.1479 | 8 | 4647 (1818/2829) | Recessive | | Random | 0.886 (0.406-1.930) | 0.76 | 42.8 | 0.093 |  |  |  |
| MMP2 | rs243865 | TvsC | Gastric | Asian | 0.1409 | 6 | 4206 (1604/2602) | Allelic | | Random | 0.670 (0.429-1.047) | 0.079 | 89.0 | 0 |  |  |  |
| MMP2 | rs243865 | TvsC | Gastric | Asian | 0.1409 | 6 | 4206 (1604/2602) | Dominant | | Random | 0.623 (0.393-0.987) | 0.044 | 87.7 | 0 |  |  |  |
| MMP2 | rs243865 | TvsC | Gastric | Asian | 0.1409 | 6 | 4206 (1604/2602) | Recessive | | Random | 0.878 (0.297-2.596) | 0.813 | 59.1 | 0.032 |  |  |  |
| MMP2 | rs243865 | TvsC | Lung | Overall | 0.1679 | 5 | 4734 (2199/2535) | Allelic | | Random | 0.654 (0.507-0.844) | 0.001 | 72.1 | 0.006 | ACC | 0.045 | moderate |
| MMP2 | rs243865 | TvsC | Lung | Overall | 0.1679 | 5 | 4734 (2199/2535) | Dominant | | Random | 0.613 (0.457-0.823) | 0.001 | 73.8 | 0.004 | ACC | 0.07 | weak |
| MMP2 | rs243865 | TvsC | Lung | Overall | 0.1679 | 5 | 4734 (2199/2535) | Recessive | | Fixed | 0.646 (0.417-1.001) | 0.051 | 0.0 | 0.908 |  |  |  |
| MMP2 | rs243865 | TvsC | Lung | Asian | 0.1667 | 3 | 4254 (1909/2345) | Allelic | | Fixed | 0.534 (0.468-0.610) | < 0.001 | 0.0 | 0.837 | AAA | 5.6E-07 | strong |
| MMP2 | rs243865 | TvsC | Lung | Asian | 0.1667 | 3 | 4254 (1909/2345) | Dominant | | Fixed | 0.484 (0.417-0.561) | < 0.001 | 0.0 | 0.864 | AAA | 2.79E-10 | strong |
| MMP2 | rs243865 | TvsC | Lung | Asian | 0.1667 | 3 | 4254 (1909/2345) | Recessive | | Fixed | 0.616 (0.385-0.985) | 0.043 | 0.0 | 0.944 | CAC | 0.688 | weak |
| MMP2 | rs1053605 | TvsC | Lung | Overall | 0.1917 | 5 | 4426 (2165/2261) | Allelic | | Random | 0.885 (0.697-1.123) | 0.314 | 66.3 | 0.018 |  |  |  |
| MMP2 | rs1053605 | TvsC | Lung | Overall | 0.1917 | 5 | 4426 (2165/2261) | Dominant | | Random | 0.899 (0.633-1.276) | 0.551 | 78.7 | 0.001 |  |  |  |
| MMP2 | rs1053605 | TvsC | Lung | Overall | 0.1917 | 5 | 4426 (2165/2261) | Recessive | | Fixed | 0.740 (0.531-1.030) | 0.074 | 0.0 | 0.65 |  |  |  |
| MMP2 | rs1053605 | TvsC | Lung | Caucasian | 0.1243 | 3 | 1805 (1037/768) | Allelic | | Fixed | 1.080 (0.884-1.320) | 0.452 | 34.6 | 0.216 |  |  |  |
| MMP2 | rs1053605 | TvsC | Lung | Caucasian | 0.1243 | 3 | 1805 (1037/768) | Dominant | | Fixed | 1.183 (0.945-1.482) | 0.143 | 40.1 | 0.189 |  |  |  |
| MMP2 | rs1053605 | TvsC | Lung | Caucasian | 0.1243 | 3 | 1805 (1037/768) | Recessive | | Fixed | 0.554 (0.289-1.063) | 0.076 | 0.0 | 0.529 |  |  |  |
| MMP2 | rs243865 | TvsC | Lymphoma | Overall | 0.1423 | 3 | 1215 (565/650) | Allelic | | Random | 1.735 (0.860-3.501) | 0.124 | 82.8 | 0.003 |  |  |  |
| MMP2 | rs243866 | TvsC | Lymphoma | Overall | 0.1423 | 3 | 1215 (565/650) | Dominant | | Random | 1.826 (0.924-3.606) | 0.083 | 73.3 | 0.023 |  |  |  |
| MMP2 | rs243867 | TvsC | Lymphoma | Overall | 0.1423 | 3 | 1215 (565/650) | Recessive | | Fixed | 1.848 (0.990-3.449) | 0.054 | 56.6 | 0.1 |  |  |  |
| MMP2 | rs243865 | TvsC | Nasopharyngeal | Asian | 0.1083 | 3 | 2946 (1381/1565) | Allelic | | Random | 0.716 (0.506-1.014) | 0.06 | 71.1 | 0.032 |  |  |  |
| MMP2 | rs243865 | TvsC | Nasopharyngeal | Asian | 0.1083 | 3 | 2946 (1381/1565) | Dominant | | Random | 0.686 (0.492-0.957) | 0.026 | 63.0 | 0.067 | BCC | 0.47 | weak |
| MMP2 | rs243865 | TvsC | Nasopharyngeal | Asian | 0.1083 | 3 | 2946 (1381/1565) | Recessive | | Fixed | 0.978 (0.415-2.305) | 0.96 | 0.0 | 0.441 |  |  |  |
| MMP2 | rs243865 | TvsC | Oral | Asian | 0.1689 | 4 | 3860 (1835/2025) | Allelic | | Random | 0.861 (0.539-1.373) | 0.529 | 92.1 | 0 |  |  |  |
| MMP2 | rs243865 | TvsC | Oral | Asian | 0.1689 | 4 | 3860 (1835/2025) | Dominant | | Random | 0.849 (0.512-1.407) | 0.525 | 91.1 | 0 |  |  |  |
| MMP2 | rs243865 | TvsC | Oral | Asian | 0.1689 | 4 | 3860 (1835/2025) | Recessive | | Random | 0.870 (0.322-2.350) | 0.783 | 76.5 | 0.005 |  |  |  |
| MMP2 | rs243865 | TvsC | Prostate | Overall | 0.2044 | 6 | 1433 (699/734) | Allelic | | Fixed | 1.191 (0.995-1.426) | 0.057 | 37.4 | 0.157 |  |  |  |
| MMP2 | rs243865 | TvsC | Prostate | Overall | 0.2044 | 6 | 1433 (699/734) | Dominant | | Fixed | 1.365 (1.094-1.703) | 0.006 | 7.1 | 0.371 | BAA | 0.122 | moderate |
| MMP2 | rs243865 | TvsC | Prostate | Overall | 0.2044 | 6 | 1433 (699/734) | Recessive | | Fixed | 0.849 (0.561-1.284) | 0.437 | 6.6 | 0.374 |  |  |  |
| MMP2 | rs243865 | TvsC | Prostate | Asian | 0.1535 | 3 | 732 (341/391) | Allelic | | Fixed | 1.480 (1.131-1.936) | 0.004 | 0.0 | 0.868 | BAA | 0.13 | moderate |
| MMP2 | rs243865 | TvsC | Prostate | Asian | 0.1535 | 3 | 732 (341/391) | Dominant | | Fixed | 1.657 (1.207-2.276) | 0.002 | 0.0 | 0.962 | BAA | 0.114 | moderate |
| MMP2 | rs243865( | TvsC | Prostate | Asian | 0.1535 | 3 | 732 (341/391) | Recessive | | Fixed | 1.247 (0.600-2.591) | 0.554 | 0.0 | 0.59 |  |  |  |
| MMP7 | rs11568818 | CvsT | Bladder | Overall | 0.291 | 4 | 2377 (1169/1208) | Allelic | | Fixed | 1.204 (1.055-1.374) | 0.006 | 0.0 | 0.405 | AAA | 0.1 | strong |
| MMP7 | rs11568818 | CvsT | Bladder | Overall | 0.291 | 4 | 2377 (1169/1208) | Dominant | | Fixed | 1.135 (0.942-1.368) | 0.184 | 1.9 | 0.383 |  |  |  |
| MMP7 | rs11568818 | CvsT | Bladder | Overall | 0.291 | 4 | 2377 (1169/1208) | Recessive | | Fixed | 1.538 (1.198-1.974) | 0.001 | 0.0 | 0.696 | BAA | 0.032 | strong |
| MMP7 | rs11568818 | CvsT | Bladder | Asian | 0.2651 | 3 | 1938 (929/1009) | Allelic | | Fixed | 1.229 (1.056-1.431) | 0.008 | 23.7 | 0.269 | AAA | 0.131 | strong |
| MMP7 | rs11568818 | CvsT | Bladder | Asian | 0.2651 | 3 | 1938 (929/1009) | Dominant | | Fixed | 1.175 (0.952-1.450) | 0.134 | 22.4 | 0.276 |  |  |  |
| MMP7 | rs11568818 | CvsT | Bladder | Asian | 0.2651 | 3 | 1938 (929/1009) | Recessive | | Fixed | 1.560 (1.166-2.087) | 0.003 | 0.0 | 0.494 | BAA | 0.116 | moderate |
| MMP7 | rs11568818 | CvsT | Breast | Asian | 0.0911 | 3 | 4876 (2411/2465) | Allelic | | Random | 1.160 (0.894-1.505) | 0.263 | 67.4 | 0.047 |  |  |  |
| MMP7 | rs11568818 | CvsT | Breast | Asian | 0.0911 | 3 | 4876 (2411/2465) | Dominant | | Fixed | 1.153 (0.985-1.349) | 0.077 | 20.9 | 0.282 |  |  |  |
| MMP7 | rs11568818 | CvsT | Breast | Asian | 0.0911 | 3 | 4876 (2411/2465) | Recessive | | Random | 1.475 (0.616-3.530) | 0.383 | 68.7 | 0.041 |  |  |  |
| MMP7 | rs11568818 | CvsT | Cervical | Asian | 0.2896 | 3 | 1179 (597/582) | Allelic | | Fixed | 1.372 (1.148-1.640) | 0.001 | 0.0 | 0.583 | BAA | 0.012 | strong |
| MMP7 | rs11568818 | CvsT | Cervical | Asian | 0.2896 | 3 | 1179 (597/582) | Dominant | | Fixed | 1.381 (1.088-1.753) | 0.008 | 52.8 | 0.12 | BCC | 0.168 | weak |
| MMP7 | rs11568818 | CvsT | Cervical | Asian | 0.2896 | 3 | 1179 (597/582) | Recessive | | Fixed | 1.664 (1.175-2.357) | 0.004 | 52.7 | 0.121 | BCA | 0.22 | weak |
| MMP7 | rs11568818 | CvsT | Colorectal | Overall | 0.2734 | 10 | 4377 (2093/2284) | Allelic | | Random | 0.953 (0.794-1.144) | 0.606 | 57.4 | 0.012 |  |  |  |
| MMP7 | rs11568818 | CvsT | Colorectal | Overall | 0.2734 | 10 | 4377 (2093/2284) | Dominant | | Fixed | 1.009 (0.873-1.166) | 0.907 | 38.1 | 0.104 |  |  |  |
| MMP7 | rs11568818 | CvsT | Colorectal | Overall | 0.2734 | 10 | 4377 (2093/2284) | Recessive | | Random | 0.908 (0.582-1.417) | 0.671 | 71.2 | 0.002 |  |  |  |
| MMP7 | rs11568818 | CvsT | Colorectal | Caucasian | 0.437 | 3 | 1719 (838/881) | Allelic | | Random | 1.240 (0.966-1.592) | 0.092 | 58.2 | 0.091 |  |  |  |
| MMP7 | rs11568818 | CvsT | Colorectal | Caucasian | 0.437 | 3 | 1719 (838/881) | Dominant | | Fixed | 1.196 (0.973-1.470) | 0.089 | 50.1 | 0.135 |  |  |  |
| MMP7 | rs11568818 | CvsT | Colorectal | Caucasian | 0.437 | 3 | 1719 (838/881) | Recessive | | Fixed | 1.208 (0.960-1.519) | 0.106 | 51.0 | 0.13 |  |  |  |
| MMP7 | rs11568818 | CvsT | Colorectal | Asian | 0.1292 | 5 | 2214 (1045/1169) | Allelic | | Fixed | 0.771 (0.629-0.945) | 0.012 | 0.0 | 0.566 | BAA | 0.202 | weak |
| MMP7 | rs11568818 | CvsT | Colorectal | Asian | 0.1292 | 5 | 2214 (1045/1169) | Dominant | | Fixed | 0.814 (0.639-1.035) | 0.094 | 5.9 | 0.373 |  |  |  |
| MMP7 | rs11568818 | CvsT | Colorectal | Asian | 0.1292 | 5 | 2214 (1045/1169) | Recessive | | Fixed | 0.450 (0.256-0.790) | 0.005 | 12.6 | 0.285 | CAC | 0.546 | weak |
| MMP7 | rs11568818 | CvsT | Gastric | Overall | 0.3058 | 9 | 3592 (1463/2129) | Allelic | | Random | 1.096 (0.834-1.441) | 0.511 | 73.4 | 0 |  |  |  |
| MMP7 | rs11568818 | CvsT | Gastric | Overall | 0.3058 | 9 | 3592 (1463/2129) | Dominant | | Random | 1.072 (0.742-1.548) | 0.712 | 71.4 | 0 |  |  |  |
| MMP7 | rs11568818 | CvsT | Gastric | Overall | 0.3058 | 9 | 3592 (1463/2129) | Recessive | | Random | 1.297 (0.846-1.988) | 0.233 | 54.2 | 0.032 |  |  |  |
| MMP7 | rs11568818 | CvsT | Gastric | Asian | 0.291 | 7 | 3151 (1249/1902) | Allelic | | Random | 1.177 (0.857-1.615) | 0.314 | 73.8 | 0.001 |  |  |  |
| MMP7 | rs11568818 | CvsT | Gastric | Asian | 0.291 | 7 | 3151 (1249/1902) | Dominant | | Random | 1.191 (0.807-1.756) | 0.379 | 67.4 | 0.005 |  |  |  |
| MMP7 | rs11568818 | CvsT | Gastric | Asian | 0.291 | 7 | 3151 (1249/1902) | Recessive | | Random | 1.467 (0.830-2.591) | 0.187 | 62.7 | 0.02 |  |  |  |
| MMP7 | rs11568818 | CvsT | Lung | Overall | 0.0613 | 3 | 1867 (733/1134) | Allelic | | Random | 1.701 (0.976-2.966) | 0.061 | 63.0 | 0.067 |  |  |  |
| MMP7 | rs11568818 | CvsT | Lung | Overall | 0.0613 | 3 | 1867 (733/1134) | Dominant | | Random | 1.676 (0.962-2.922) | 0.068 | 58.4 | 0.09 |  |  |  |
| MMP7 | rs11568818 | CvsT | Lung | Overall | 0.0613 | 3 | 1867 (733/1134) | Recessive | | Random | 1.824 (0.820-4.058) | 0.141 | 0.0 | 0.657 |  |  |  |
| MMP9 | rs3918242 | TvsC | Bladder | Caucasian | 0.1663 | 3 | 1764 (901/863) | Allelic | | Fixed | 0.946 (0.791-1.133) | 0.549 | 24.0 | 0.268 |  |  |  |
| MMP9 | rs3918242 | TvsC | Bladder | Caucasian | 0.1663 | 3 | 1764 (901/863) | Dominant | | Fixed | 0.940 (0.764-1.156) | 0.555 | 31.0 | 0.235 |  |  |  |
| MMP9 | rs3918242 | TvsC | Bladder | Caucasian | 0.1663 | 3 | 1764 (901/863) | Recessive | | Fixed | 0.932 (0.543-1.598) | 0.797 | 0.0 | 0.878 |  |  |  |
| MMP9 | rs3918242 | TvsC | Breast | Overall | 0.1967 | 6 | 3316 (1656/1660) | Allelic | | Fixed | 1.281 (1.134-1.447) | < 0.001 | 29.2 | 0.216 | AAA | 0.001 | strong |
| MMP9 | rs3918242 | TvsC | Breast | Overall | 0.1967 | 6 | 3316 (1656/1660) | Dominant | | Fixed | 1.236 (1.065-1.434) | 0.005 | 21.8 | 0.27 | AAC | 0.09 | weak |
| MMP9 | rs3918242 | TvsC | Breast | Overall | 0.1967 | 6 | 3316 (1656/1660) | Recessive | | Fixed | 1.681 (1.279-2.209) | < 0.001 | 0.0 | 0.723 | BAA | 0.017 | strong |
| MMP9 | rs3918242 | TvsC | Breast | Asian | 0.3042 | 3 | 1196 (601/595) | Allelic | | Fixed | 1.501 (1.263-1.785) | < 0.001 | 0.0 | 0.731 | BAC | 0.0002 | moderate |
| MMP9 | rs3918242 | TvsC | Breast | Asian | 0.3042 | 3 | 1196 (601/595) | Dominant | | Fixed | 1.526 (1.207-1.930) | < 0.001 | 0.0 | 0.628 | BAA | 0.018 | strong |
| MMP9 | rs3918242 | TvsC | Breast | Asian | 0.3042 | 3 | 1196 (601/595) | Recessive | | Fixed | 1.710 (1.262-2.317) | 0.001 | 0.0 | 0.917 | BAA | 0.049 | strong |
| MMP9 | rs3918242 | TvsC | Colorectal | Overall | 0.1303 | 6 | 2396 (1072/1324) | Allelic | | Random | 1.051 (0.804-1.375) | 0.714 | 56.7 | 0.042 |  |  |  |
| MMP9 | rs3918242 | TvsC | Colorectal | Overall | 0.1303 | 6 | 2396 (1072/1324) | Dominant | | Random | 1.056 (0.779-1.429) | 0.727 | 57.9 | 0.036 |  |  |  |
| MMP9 | rs3918242 | TvsC | Colorectal | Overall | 0.1303 | 6 | 2396 (1072/1324) | Recessive | | Fixed | 1.507 (0.830-2.734) | 0.178 | 0.0 | 0.46 |  |  |  |
| MMP9 | rs3918242 | TvsC | Colorectal | Asian | 0.1344 | 5 | 2061 (945/1116) | Allelic | | Fixed | 1.039 (0.754-1.432) | 0.816 | 65.3 | 0.021 |  |  |  |
| MMP9 | rs3918242 | TvsC | Colorectal | Asian | 0.1344 | 5 | 2061 (945/1116) | Dominant | | Random | 1.032 (0.718-1.483) | 0.866 | 65.8 | 0.02 |  |  |  |
| MMP9 | rs3918242 | TvsC | Colorectal | Asian | 0.1344 | 5 | 2061 (945/1116) | Recessive | | Fixed | 1.422 (0.802-2.520) | 0.228 | 0.0 | 0.462 |  |  |  |
| MMP9 | rs17576 | GvA | Colorectal | Asian | 0.3331 | 3 | 1314 (559/755) | Allelic | | Random | 0.796 (0.566-1.121) | 0.192 | 75.0 | 0.018 |  |  |  |
| MMP9 | rs17576 | GvA | Colorectal | Asian | 0.3331 | 3 | 1314 (559/755) | Dominant | | Random | 0.717 (0.455-1.130) | 0.151 | 75.9 | 0.016 |  |  |  |
| MMP9 | rs17576 | GvA | Colorectal | Asian | 0.3331 | 3 | 1314 (559/755) | Recessive | | Fixed | 0.821 (0.566-1.189) | 0.296 | 0.0 | 0.433 |  |  |  |
| MMP9 | rs3918242 | TvsC | Esophageal | Asian | 0.5586 | 3 | 844 (426/418) | Allelic | | Random | 0.948 (0.635-1.416) | 0.794 | 58.1 | 0.092 |  |  |  |
| MMP9 | rs3918242 | TvsC | Esophageal | Asian | 0.5586 | 3 | 844 (426/418) | Dominant | | Random | 0.825 (0.417-1.632) | 0.581 | 65.8 | 0.054 |  |  |  |
| MMP9 | rs3918242 | TvsC | Esophageal | Asian | 0.5586 | 3 | 844 (426/418) | Recessive | | Fixed | 0.904 (0.619-1.322) | 0.603 | 0.0 | 0.423 |  |  |  |
| MMP9 | rs3918242 | TvsC | Gastric | Overall | 0.2145 | 5 | 1520 (697/823) | Allelic | | Fixed | 1.174 (0.985-1.400) | 0.073 | 42.0 | 0.142 |  |  |  |
| MMP9 | rs3918242 | TvsC | Gastric | Overall | 0.2145 | 5 | 1520 (697/823) | Dominant | | Random | 1.103 (0.767-1.586) | 0.596 | 60.2 | 0.04 |  |  |  |
| MMP9 | rs3918242 | TvsC | Gastric | Overall | 0.2145 | 5 | 1520 (697/823) | Recessive | | Fixed | 1.526 (0.965-2.412) | 0.07 | 0.0 | 0.761 |  |  |  |
| MMP9 | rs3918242 | TvsC | Gastric | Asian | 0.2385 | 3 | 1128 (539/589) | Allelic | | Random | 1.203 (0.875-1.654) | 0.254 | 61.1 | 0.076 |  |  |  |
| MMP9 | rs3918242 | TvsC | Gastric | Asian | 0.2385 | 3 | 1128 (539/589) | Dominant | | Random | 1.176 (0.692-2.000) | 0.549 | 76.8 | 0.014 |  |  |  |
| MMP9 | rs3918242 | TvsC | Gastric | Asian | 0.2385 | 3 | 1128 (539/589) | Recessive | | Fixed | 1.612 (1.000-2.598) | 0.05 | 0.0 | 0.551 | CAA | 0.712 | weak |
| MMP9 | rs3918242 | TvsC | Hepatocellular | Overall | 0.1589 | 3 | 1280 (657/623) | Allelic | | Random | 1.035 (0.609-1.758) | 0.898 | 78.7 | 0.009 |  |  |  |
| MMP9 | rs3918242 | TvsC | Hepatocellular | Overall | 0.1589 | 3 | 1280 (657/623) | Dominant | | Fixed | 0.933 (0.720-1.208) | 0.597 | 53.7 | 0.115 |  |  |  |
| MMP9 | rs3918242 | TvsC | Hepatocellular | Overall | 0.1589 | 3 | 1280 (657/623) | Recessive | | Fixed | 1.973 (1.068-3.645) | 0.03 | 48.1 | 0.165 | CBA | 0.749 | weak |
| MMP9 | rs3918242 | TvsC | Lung | Overall | 0.141 | 5 | 2980 (1539/1447) | Allelic | | Random | 0.754 (0.570-0.999) | 0.049 | 63.3 | 0.028 | BCA | 0.537 | weak |
| MMP9 | rs3918242 | TvsC | Lung | Overall | 0.141 | 5 | 2980 (1539/1447) | Dominant | | Random | 0.763 (0.552-1.055) | 0.102 | 66.6 | 0.018 |  |  |  |
| MMP9 | rs3918242 | TvsC | Lung | Overall | 0.141 | 5 | 2980 (1539/1447) | Recessive | | Fixed | 0.355 (0.177-0.712) | 0.004 | 42.1 | 0.159 | CCA | 0.639 | weak |
| MMP9 | rs3918242 | TvsC | Lung | Caucasian | 0.1496 | 3 | 1890 (1051/839) | Allelic | | Fixed | 0.798(0.661-0.962） | 0.018 | 0.0 | 0.502 | BAA | 0.276 | weak |
| MMP9 | rs3918242 | TvsC | Lung | Caucasian | 0.1496 | 3 | 1890 (1051/839) | Dominant | | Fixed | 0.853 (0.6921.051） | 0.135 | 0.0 | 0.561 |  |  |  |
| MMP9 | rs3918242 | TvsC | Lung | Caucasian | 0.1496 | 3 | 1890 (1051/839) | Recessive | | Fixed | 0.257 (0.118-0.561) | 0.001 | 0.0 | 0.615 | CAC | 0.595 | weak |
| MMP9 | rs3918242 | TvsC | Oral | Overall | 0.1406 | 3 | 1545 (770/775) | Allelic | | Fixed | 1.309 (1.078-1.589) | 0.007 | 26.8 | 0.255 | BAA | 0.118 | moderate |
| MMP9 | rs3918242 | TvsC | Oral | Overall | 0.1406 | 3 | 1545 (770/775) | Dominant | | Random | 1.225 (0.825-1.819) | 0.313 | 64.8 | 0.058 |  |  |  |
| MMP9 | rs3918242 | TvsC | Oral | Overall | 0.1406 | 3 | 1545 (770/775) | Recessive | | Fixed | 3.497 (1.812-6.749) | < 0.001 | 0.0 | 0.717 | CAA | 0.383 | weak |

Abbreviations: A, adenine; C, cytosine; G, guanine; T, thymine; OR, odds ratio; CI, confidence interval; MAF, minor allelic frequency in control; FPRP, false-positive report probability.

^a^ Allelics: Minor allelic (bold) vs major allelic.

^b^ Venice criteria grades are for amount of evidence, replication of the association and protection from bias.

^c^ The prior probability of FPRP is 0.05, and the FPRP level of noteworthiness is 0.20.
